# Interactive multi-model fault diagnosis method of switched reluctance motor based on low delay anti-interference

**DOI:** 10.1371/journal.pone.0270536

**Published:** 2023-01-31

**Authors:** Yongqin Zhou, Chongchong Wang, Yongchao Wang, Yubin Wang, Yujia Chang

**Affiliations:** 1 School of Electrical and Electronic Engineering, Harbin University of Science and Technology, Harbin, China; 2 State Grid Harbin Electric Power Supply Company, Harbin, China; Beijing Institute of Technology, CHINA

## Abstract

Given fault false alarm and fault control failure caused by the decrease of fault identification accuracy and fault delay of Switched Reluctance Motor (SRM) power converter in complex working conditions, a method based on the Interactive Multi-Model (IMM) algorithm was proposed in this paper. Besides, the corresponding equivalent circuit models were established according to the different working states of the SRM power converter. The Kalman filter was employed to estimate the state of the model, and the fault detection and location were realized depending on the residual signal. Additionally, a transition probability correction function of the IMM was constructed using the difference of the n-th order to suppress the influence of external disturbance on the fault diagnosis accuracy. Concurrently, a model jump threshold was introduced to reduce delay when the matched model was switched, so as to realize the rapid separation of faults and effective fault control. The simulation and experiment results demonstrate that the IMM algorithm based on low delay anti-interference can effectively reduce the influence of complex working conditions, improve the anti-interference ability of SRM power converter fault diagnosis, and identify fault information accurately and quickly.

## Introduction

A switched reluctance motor drive system (SRD) is a typical mechatronic device, which is mainly composed of a switched reluctance motor (SRM) and a power converter [1]. SRM is widely used in electric vehicles, household appliances, aerospace, and other fields due to its simple structure, low cost, strong reliability, and wide speed regulation range [2–4]. Power converter, as an essential part of SRD, undertakes the task of delivering power to SRM [5]. Particularly, the main switching tube of the power converter frequently operates in a high-frequency switching state and a high-temperature environment and is easily subjected to voltage or current impact during the switching process. As a result, the switching device loss increases, and the heat is severe, making it most prone to failure. If the fault is not detected and removed in time, the failure and paralysis of the entire system would be induced [6, 7]. Therefore, the research on the fault diagnosis method of SRM power converter has attracted the attention of worldwide researchers. At present, the commonly used fault diagnosis methods for SRM power converter majorly comprise the current detection method, voltage detection method, and pattern identification method.

Fault diagnosis methods based on current detection and voltage detection generally obtain system status information by increasing the number of sensors and identify faults by determining whether the sensor output signal exceeds the threshold [8–10]. These two methods are simple and easy to implement. However, it is difficult to find potential early failures of the system, boosting the cost of the system and the complexity of the main circuit structure.

Compared with the current detection method and the voltage detection method, the mode identification method completes the fault identification by collecting and mathematically transforming the fault current, voltage, or other fault signals of the main circuit of the system to obtain the fault characteristic quantity [11, 12]. In recent years, some fault diagnosis methods based on the mode identification method have achieved better results, such as wavelet transform [13, 14], fast Fourier transform (FFT) [15, 16], empirical mode decomposition (EMD) [17–19], variational mode decomposition (VMD) [20, 21], and others [22–24]. With the wavelet packet node energy dispersion as the fault feature, the open-circuit and short-circuit faults of the power converter are detected by judging the change in the phase current node energy dispersion before and after the fault [25]. However, the calculation of this method is relatively complicated, the effect is not ideal at low speed, and the short-circuit fault devices cannot be distinguished. Additionally, the dq-axis current running trajectories of open-circuit and short-circuit faults are analyzed [26]. If the detected current amplitude is different from normal operation, a fault occurs, and the fault diagnosis of the SRM power converter is realized. The method is computationally complex and cannot achieve fault location. The performance of the SRM drive system before and after open-circuit and short-circuit faults is predicted using a model based on neural network and genetic algorithm [27]. Nonetheless, these two algorithms are complex and computationally expensive, and present severe fault diagnosis delay, making them difficult to be implemented. Later, an online fault diagnosis scheme based on freewheeling current is provided to analyze the influence of different faults on the upper and lower freewheeling currents and extract fault characteristic coefficients with the idea of digitization for fault diagnosis [28]. However, this method performs fault diagnosis for each phase and requires two current sensors, leading to an increase in the system volume and cost. Moreover, the diagnosis time is longer than one phase current cycle, resulting in a long diagnosis delay. The current spectrum characteristics of the bus before and after the fault are analyzed by defining three different bus positions, and the fast Fourier transform algorithm is combined with the Blackman window interpolation [29]. The use of a single current sensor to detect the open circuit fault diagnosis of the power tube is completed as the fault feature quantity. Unfortunately, the algorithm of this scheme is complex, and the fence effect of the fast Fourier transform algorithm inevitably produces spectral energy leakage, disturbing the fault information and influencing the fault diagnosis accuracy. Furthermore, a fault diagnosis method based on State Inverse Solution (SIS) is designed to rearrange the position of traditional current sensors and inversely solve the phase current according to the established switching tube state solution model, which is the same as the real value [30]. For comparison, the fault diagnosis is realized through the established fault table in the combination of the switch tube driving signal. However, a systematic error is generated when the current changes abruptly. A fault diagnosis method is also proposed based on the combination of Variational Mode Decomposition and Multi-scale Permutation Entropy (VMD-MPE) [31]. Specifically, it is decomposed to obtain several eigenmode components, the average value of the permutation entropy of the multi-scale effective modal components is taken as the eigenvector, and the support vector machine classifier is inputted for fault identification, so as to effectively weaken the influence of noise and improve the fault accuracy. The biggest limitation of the scheme is that it cannot handle the occurrence of sudden signals. False alarms will appear when the current signal jumps, affecting the accuracy of fault diagnosis and impeding it from locating the faulty device. The above literature suggests that the mode identification method can complete the fault diagnosis of SRM power converters well without increasing the hardware cost. However, the above-mentioned literature only analyzes a single fault, identifies the type of fault, and cannot distinguish the components where the fault occurs. Meanwhile, the algorithm is complex, the amount of calculation is large, a delay exists in fault diagnosis, and the algorithm is easily interfered with by the noise of the input signal, influencing the diagnostic accuracy. Particularly, the current in the power converter will change drastically when the SRM operates in complex working conditions, leading to false alarms in fault detection and affecting the accuracy of fault diagnosis.

The theory of Interacting Multiple Model (IMM) has been emphasized in the research of multi-fault diagnosis technology. IMM is a multi-model algorithm proposed by Israeli scientist Barsholm in 1988. It assumes that the jump between models is guided by a finite-length Markov chain and effectively controls the number of parallel filters while maintaining information interaction [32]. By establishing the corresponding fault models for the possible faults of the system, they are incorporated into the total model set as part of the system, and then the IMM algorithm is adopted to find the matching model under the current working state of the system. If the system fails, fault detection and fault separation are performed according to matching models [33]. In the existing research, no scientific achievements have been discovered in applying the IMM algorithm to the multi-fault diagnosis of SRM power converter; the IMM algorithm has already had certain development and applications in other fault diagnosis fields. Additionally, a fault diagnosis method based on the combination of IMM and Unscented Kalman filter (UKF) is proposed for the sudden failure of the electromechanical actuator of the aircraft flight control system [34]. The filter interactive input stage assists in suppressing the influence of residual deviation caused by noise on the Kalman filter, quickly locating faults, and improving the rapidity and accuracy of fault diagnosis. IMM is applied to separate the parameter faults of the vehicle’s vertical shock absorber to different degrees [35]. The results demonstrate that inaccurate model transition probability reduces the accuracy of fault diagnosis results, realizes rapid fault location, and intuitively judges vibration-damping the degree of failure of the device. Additionally, the Particle Filter (PF) method and the IMM method are introduced for fault diagnosis and adaptive estimation of the wind turbine pitch system including various faults of the sensor; the model transition probability of the IMM in the non-mode switching stage is adaptively modified to improve the state estimation accuracy of the method; the model probabilities are corrected using an inversion strategy in the mode switching stage to improve the diagnosis speed and reduce the model mismatch, contributing to improving the accuracy of the diagnosis. IMM is improved with the asynchronous sensor fusion to complete the fault detection and multi-fault location separation of open-circuit faults of multiple IGBT switches of traction inverters of high-speed trains [36]. The IMM algorithm is introduced into the condition monitoring and fault diagnosis of railway vehicles to visually separate the fault location and reduce the probability of fault misjudgment [37]. Besides, an IMM based on low inertia and anti-noise (LN-IMM) is proposed [38]. The multi-fault diagnosis of lithium-ion batteries can be realized by combining with strong tracking Kalman filter (STKF). LN-IMM can not only efficiently complete the state estimation of lithium-ion batteries but also enhance the accuracy of fault diagnosis and reduce the delay time of diagnosis. The above literature reveals that IMM has a solid theoretical foundation and better robustness to fault signals. Moreover, it has better performance in fault diagnosis, prediction, and noise reduction while accurately detecting and rapidly separating fault information.

The main contributions of this article are described as follows:

1. An Interactive Multi-Model (IMM) algorithm is proposed to realize fault diagnosis of the SRM power converter.
2. The corresponding equivalent circuit model is established according to the different working states of the SRM power converter. STKF is adopted to estimate the state of the model. The direct detection and location of multiple faults of the SRM power converter are realized based on the residual signal output by the Kalman filter.
3. The N-order difference based on model probability is employed to suppress the influence of complex working conditions on fault detection accuracy and achieve accurate fault information detection.
4. A model jump threshold is introduced to shorten the delay of the matching model during switching and overcome the delay in fault diagnosis.

## Methods

### Establishing an equivalent model for the SRM power converter

The SRM power converter serves as the central mechanism of SRD and is the link for SRM in realizing electromechanical energy conversion. It is similar to the inverter in a vector control asynchronous motor system, and its quality is very important to SRD [39]. The power main switching tubes in the power converter are usually IGBT, which are connected in series with the phase winding of the SRM. Moreover, the on and off of the SRM phase winding and the voltage of the phase winding terminal are controlled by controlling the opening and closing states [40].

There are two key forms of power main switch failure: short-circuit failure due to overvoltage breakdown and open-circuit failure caused by loss of drive signal. The short-circuit fault of the power main switch tube in the SRM power converter results in the increase of the current of the faulty phase. In addition, the short-circuit fault can also change the freewheeling mode of the faulty phase winding of the motor, which may cause the phase winding current to enter the inductance drop zone of the winding. Braking torque aggravates the torque ripple of the motor, which seriously affects the performance of SRD. The open circuit fault of the power main switch in the SRM power converter will cause the motor to enter a phase-loss operation state, thus affecting the performance of the system [41]. Therefore, this study focused on Single-phase double-tube failure and multi-phase mixed failure of main switch tube in the asymmetric half-bridge SRM power converter.

**Fig 1:**
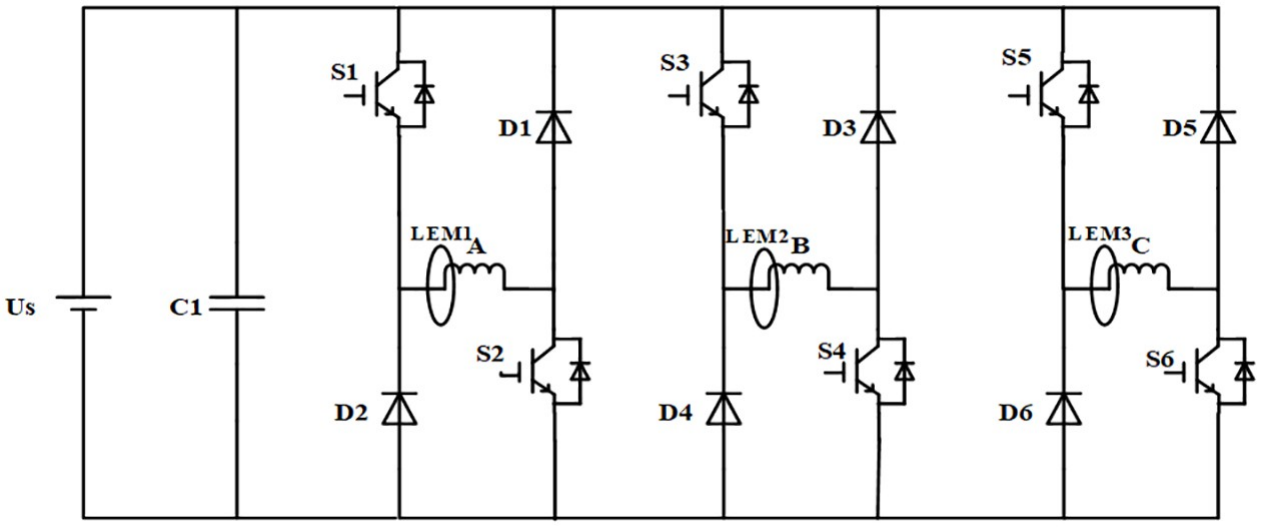
Topology of SRM power converter.

**Fig 2:**
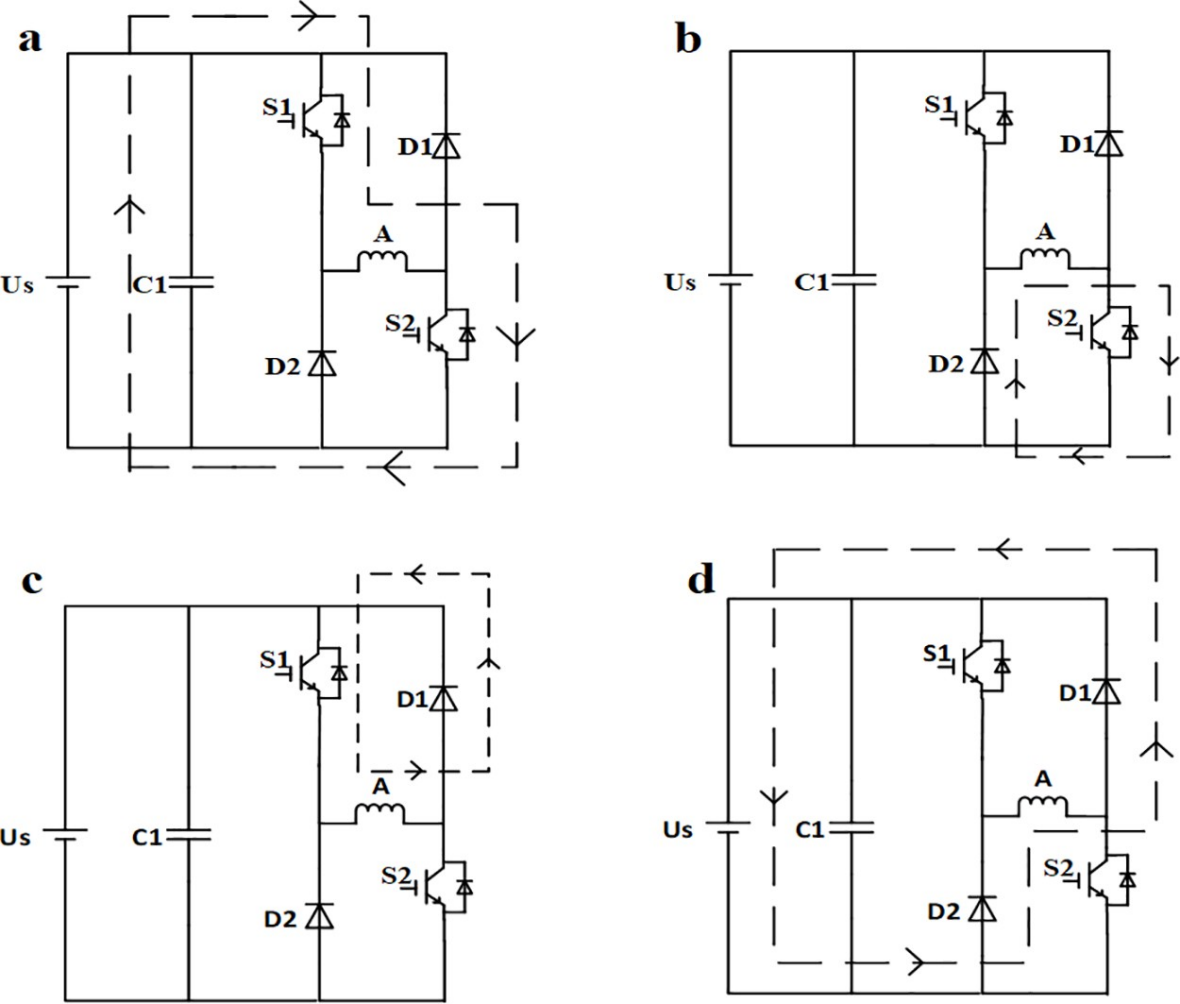
Working state of phase A. (a) Excitation ST1. (b) Lower freewheeling ST2. (c) Upper freewheeling ST3. (d) Demagnetization ST4.

The main circuit topology of the half-bridge SRM power converter is shown in Fig 1, where each phase bridge arm consists of two power switches and two freewheeling diodes. Moreover, the current sensor directly measures the winding current, in which the phase-to-phase operation is independent of each other. The two switching tubes of each phase are able to form four switching states, corresponding to four different working states of each phase. Taking phase A as an example, the four current paths are shown in Fig 2, which are the excitation, lower freewheeling, upper freewheeling and demagnetization states, respectively. For convenience, the four states were named ST1, ST2, ST3 and ST4, respectively.

In order to restrain torque ripple, reduce switching loss and iron loss, each phase adopted the soft chopping mode of chopping single tube in the conduction interval. For example, in the conduction interval of phase A, the lower tube ST2 was controlled by the position signal in order to remain on, while the upper tube ST1 was controlled by the chopping signal, and the state of phase A constantly switched between ST1 and ST2. In the turn-off interval, both tubes were turned off, and the state of phase A was ST4. Therefore, during normal operation, phase A only had three working states, namely, ST1, ST2 and ST4, for which the corresponding circuit balance equations are as follows:

$$\begin{array}{c} U_{S}=i_{a}R_{a}+L_{a}\frac{\text{d}i_{a}}{\text{d}t} \end{array}$$ (1)

$$\begin{array}{c} 0=i_{a}R_{a}+L_{a}\frac{\text{d}i_{a}}{\text{d}t} \end{array}$$ (2)

$$\begin{array}{c} -U_{S}=i_{a}R_{a}+L_{a}\frac{\text{d}i_{a}}{\text{d}t} \end{array}$$ (3)

Here, *U*_*S*_ is the power supply voltage, and *i*_*a*_, *R*_*a*_ are the current, resistance and inductance of phase winding, respectively;

The following reasonable assumptions were then made for the circuit:

1. The power switch tube is an ideal switch, that is, the forward voltage drop is zero during conducting and the resistance is infinite during blocking, so the influence of parameter nonlinearity is not considered;
2. The DC-side capacitor voltage is balanced and equal to the DC-side voltage;
3. Other components of the main circuit, such as resistors, capacitors and inductors, are ideal.

**Fig 3:**
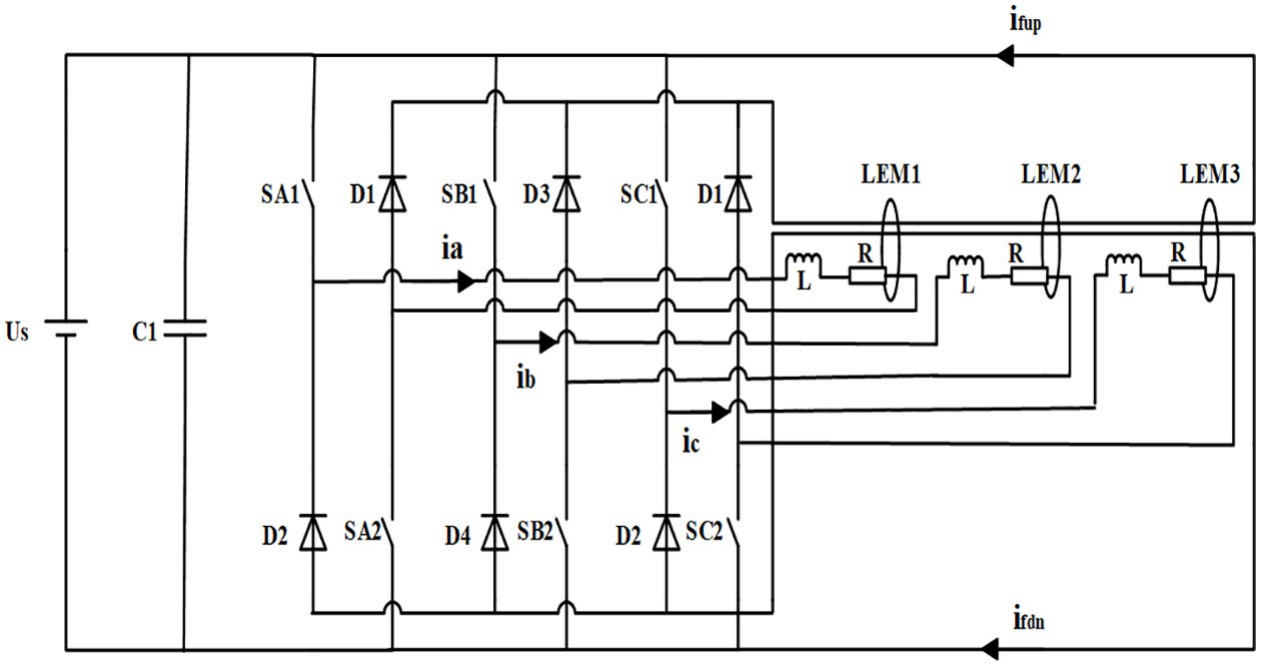
Switch equivalent circuit of the SRM power converter.

**Fig 4:**
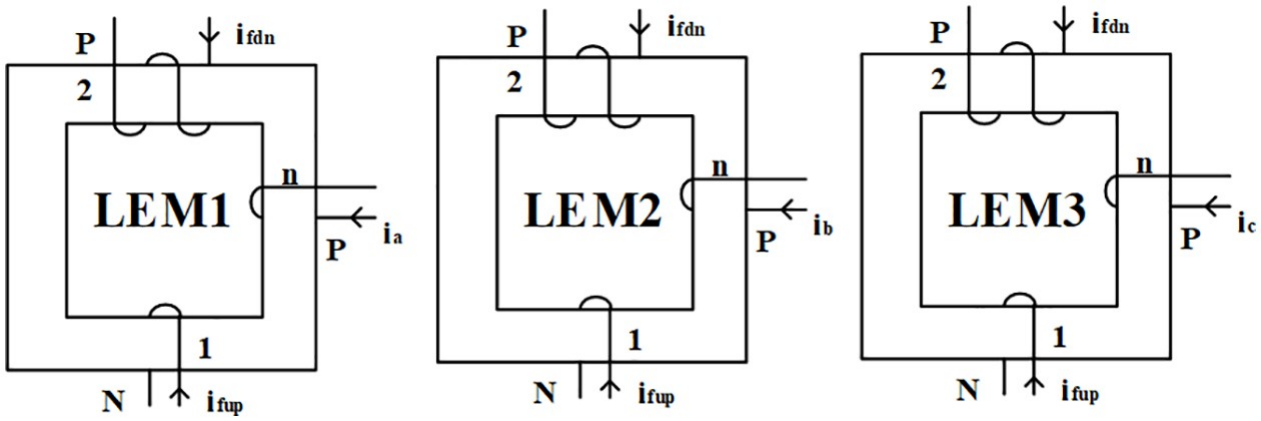
Current sensor detection mode.

Based on the above simplified assumptions, the main circuit diagram of SRM power converter can be considered equivalent to the switch equivalent circuit shown in Fig 3 by replacing the power switch devices with ideal switches, in which the current sensors *LEM*1 ∼ *LEM*3 were rearranged in order to realize the proposed fault diagnosis method and ensure the accurate detection of each phase current without affecting the normal operation of the system. Additionally, *i*_*fup*_ and *i*_*fdn*_ refer to currents of the up-freewheeling and down-freewheeling buses, respectively; Fig 4 shows the winding mode of each current sensor. “P” and “N” represent the given positive winding direction and negative winding direction, respectively. Moreover if *n*: 1: 2 is the winding turns ratio, then the output values of the current sensor are:

$$\begin{array}{c} i_{1}=ni_{a}-i_{fup}+2i_{fdn} \end{array}$$ (4)

$$\begin{array}{c} i_{2}=ni_{b}-i_{fup}+2i_{fdn} \end{array}$$ (5)

$$\begin{array}{c} i_{3}=ni_{c}-i_{fup}+2i_{fdn} \end{array}$$ (6)

A single power management switch state variable *S*_*xi*_ was defined to represent the switch states of the upper and lower switches of phase A, phase B and phase C of the bridge arm. The values of switch state variables were:

$$\begin{array}{c} S_{xi}=\{\begin{array}{cc} 1, & \text{Turn}\;\text{on}\\ 0, & \text{Turn}\;\text{off} \end{array} \end{array}$$ (7)

Here, *x* = {*A*, *B*, *C*}, *i* = {1, 2}.

Phase A was defined as an example:

$$\begin{array}{c} T_{A}=\{\begin{array}{cc} 1, & \left(S_{A1}=1\right)\wedge \left(S_{A2}=1\right)\\ 0, & \left(S_{A1}=0\right)\wedge \left(S_{A2}=1\right)|\left(S_{A1}=1\right)\wedge \left(S_{A2}=0\right)\\ -1, & \left(S_{A1}=0\right)\wedge \left(S_{A2}=0\right) \end{array} \end{array}$$ (8)

Then Eqs (1)–(3) was equivalent to:

$$\begin{array}{c} T_{A}U_{S}=i_{a}R_{a}+L_{a}\frac{\text{d}i_{a}}{\text{d}t} \end{array}$$ (9)

Similarly, the circuit balance equation corresponding to B and C phases was equivalent to:

$$\begin{array}{c} T_{B}U_{S}=i_{b}R_{b}+L_{b}\frac{\text{d}i_{b}}{\text{d}t} \end{array}$$ (10)

$$\begin{array}{c} T_{C}U_{S}=i_{c}R_{c}+L_{c}\frac{\text{d}i_{c}}{\text{d}t} \end{array}$$ (11)

And

$$\begin{array}{c} T_{B}=\{\begin{array}{cc} 1, & \left(S_{B1}=1\right)\wedge \left(S_{B2}=1\right)\\ 0, & \left(S_{B1}=0\right)\wedge \left(S_{B2}=1\right)|\left(S_{B1}=1\right)\wedge \left(S_{B2}=0\right)\\ -1, & \left(S_{B1}=0\right)\wedge \left(S_{B2}=0\right) \end{array} \end{array}$$ (12)

$$\begin{array}{c} T_{C}=\{\begin{array}{cc} 1, & \left(S_{C1}=1\right)\wedge \left(S_{C2}=1\right)\\ 0, & \left(S_{C1}=0\right)\wedge \left(S_{C2}=1\right)|\left(S_{C1}=1\right)\wedge \left(S_{C2}=0\right)\\ -1, & \left(S_{C1}=0\right)\wedge \left(S_{C2}=0\right) \end{array} \end{array}$$ (13)

*i*_*b*_, *R*_*b*_ and *L*_*b*_ were the current, resistance and inductance of phase B winding respectively; *i*_*c*_, *R*_*c*_ and *L*_*c*_ were the current, resistance and inductance of phase C winding respectively;

Eqs (9) ∼ (11) were ombined and written as the equation of state:

$$\begin{array}{c} \left[\begin{array}{c} \frac{\text{d}i_{a}}{\text{d}t}\\ \frac{\text{d}i_{b}}{\text{d}t}\\ \frac{\text{d}i_{c}}{\text{d}t} \end{array}]=[\begin{array}{ccc} -\frac{R_{a}}{L_{a}} & 0 & 0\\ 0 & -\frac{R_{b}}{L_{b}} & 0\\ 0 & 0 & -\frac{R_{c}}{L_{c}} \end{array}][\begin{array}{c} i_{a}\\ i_{b}\\ i_{c} \end{array}]+[\begin{array}{ccc} -\frac{1}{L_{a}} & 0 & 0\\ 0 & -\frac{1}{L_{b}} & 0\\ 0 & 0 & -\frac{1}{L_{c}} \end{array}][\begin{array}{c} U_{La}\\ U_{Lb}\\ U_{Lc} \end{array}\right] \end{array}$$ (14)

Here, *U*_*La*_ = *T*_*A*_*U*_*S*_, *U*_*Lb*_ = *T*_*B*_*U*_*S*_, *U*_*Lc*_ = *T*_*C*_*U*_*S*_.

### Principle of multi-fault diagnosis algorithm based on interactive multi-model estimation

#### Interaction multiple model algorithm

**Fig 5:**
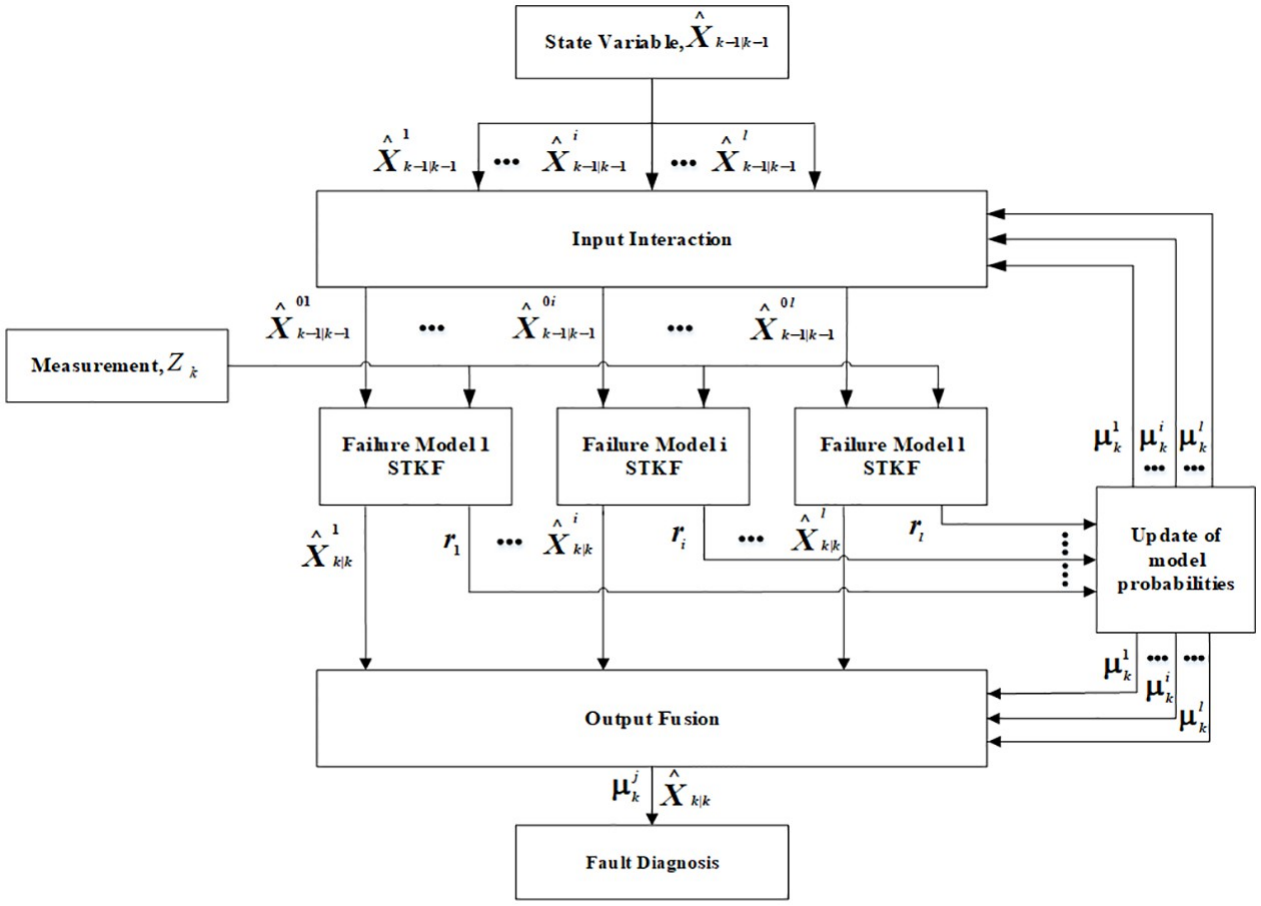
IMM algorithm flow diagram.

Fig 5 illustrates the flow chart of the IMM algorithm. IMM assumed that there are *j* fault diagnosis models which form a set {*m*_1_, *m*_2_, *m*_3_ … *m*_*l*_}. Each fault model can be represented by a Kalman filter (the filter in this paper is STKF). Each fault model will estimate the target state and calculate the failure probability of each model which is called model probability based on the input and measurement information of the system. At the output end, the matching model of the system at the current time can be calculated by information weighted fusion, which represents the current working state of the system and can realize fault detection and separation [42].

The state equation and measurement equation of the *j* fault model was elected:

$$\begin{array}{c} \left\{ \begin{array}{c} X_{k}^{j}=f_{k-1}^{j}\left(X_{k-1}^{j},\Phi_{k-1}\right)+w_{k-1}^{j}\\ Z_{k}=h_{k}^{j}\left(X_{k}^{j},\Phi_{k}\right)+v_{k} \end{array}\right. \end{array}$$ (15)

Where $X_{k-1}^{j}$ was the state variable of the system, *Z*_*k*_ was the observed variable of the system, Φ_*k*_ was the control variable of the system, *f* and *h* were nonlinear functions, $w_{k}^{j}$ was the process noise matrix of the system, and *v*_*k*_ was the measurement noise matrix of thesystem, both of which conformed to Gaussian distribution. Additionally, the variances were $Q_{k}^{j}$ and *R*_*k*_, respectively, and known. Meanwhile, each model had an independent Kalman Filter to track its state.

IMM adopted the transition probability matrix so as to control the information interaction and model conversion between various fault models. Accordingly, the transition probability matrix composed of *l* fault models in set m was expressed as:

$$\begin{array}{c} \prod ={\left\{\pi^{ij}\right\}}_{l\times l} \end{array}$$ (16)

$$\begin{array}{c} \pi^{ij}=P\left\{m\left(k\right)=m_{j}|m\left(k-1\right)=m_{i}\right\},\forall i,j\in \left\{1,2,\ldots l\right\} \end{array}$$ (17)

$$\begin{array}{c} \sum_{j=1}^{m}\pi^{ij}=1,0\leq \pi_{ij}\leq 1 \end{array}$$ (18)

where the main diagonal element of ∏ was the model probability, while the non-main diagonal element was the mixed probability.

The IMM algorithm was mainly divided into four parts:input interaction, parallel filtering, model probability calculation and fused output [43].

1. Input interaction.

Contrary to the ordinary Kalman Filter improved algorithm, IMM did not directly use the filtering result at time *k* − 1 as the input value at time *k*; instead, it interacted with the input information prior to filtering. The information following the interaction was taken as the input value of the fault model at time *k*, and the transition probability matrix guided the input information of each model according to the rules of (14) and (15). Mixing information in this manner also determined that the conversion between models was guided by the rules of the transition probability matrix:

Update of state estimate:

$$\begin{array}{c} {\hat{X}}_{k-1|k-1}^{0j}=\sum_{i=1}^{l}{\hat{X}}_{k-1|k-1}^{i}\mu_{k-1|k-1}^{ij} \end{array}$$ (19)

Update of covariance matrix:

$$\begin{array}{c} P_{k-1|k-1}^{0j}=\sum_{i=1}^{l}\mu_{k-1|k-1}^{ij}[P_{k-1|k-1}^{i}+\left({\hat{X}}_{k-1|k-1}^{i}-{\hat{X}}_{k-1|k-1}^{0j}\right){\left({\hat{X}}_{k-1|k-1}^{i}-{\hat{X}}_{k-1|k-1}^{0j}\right)}^{T}] \end{array}$$ (20)

$P_{k-1|k-1}^{0j}$ was the updated covariance matrix, ${\hat{X}}_{k-1|k-1}^{0j}$ was the updated state estimate, and $\mu_{k-1|k-1}^{ij}$ is the mixed probability of other models transferring to the *m*_*i*_ model, and its calculation formula:

$$\begin{array}{c} \left\{ \begin{array}{c} \mu_{k-1|k-1}^{ij}=\pi^{ij}\mu_{k-1}^{i}/{\bar{c}}_{j}\\ {\bar{c}}_{j}=\sum_{i=1}^{r}\pi^{ij}\mu_{k-1}^{i} \end{array}\right. \end{array}$$ (21)

Where $\mu_{k-1}^{i}$ was the model probability of the *m*_*i*_ model at the time *k* − 1.

2. Filtering process based on STKF

Each STKF in IMM worked independently and in parallel. Each STKF represented a fault model, and each STKF estimated the target state in view of the input and measurement information of the system. When the SRM power converter failed, its model parameters will change accordingly, which will be directly reflected in the sensor current. Therefore, in order to achieve the accurate detection of multiple faults of SRM power converter, having an accurate estimation of sensor current should serve as the premise. STKF generated small approximation errors when dealing with nonlinear systems while introducing a time-varying fading lactor to force the residual to be orthogonal in order to promote the robustness of the model. In this regard, it had an extremely strong tracking ability for slow and abrupt changes [44]. Accordingly, STKF was found to be very suitable as a filtering algorithm for multi-fault diagnosis of SRM power converter.

According to the functions $f_{k-1}^{j}\left(X_{k-1}^{j},\Phi_{k-1}\right)$ and $h_{k}^{j}\left(X_{k}^{j},\Phi_{k}\right)$ under the *m*_*j*_ model given by the Eq (12) and the Tailor expansion was conducted, where the first-degree term and vero-degree term were reserved, and the following was obtained:

$$\begin{array}{c} \left\{ \begin{array}{c} f_{k-1}^{j}\left(X_{k-1}^{j},\Phi_{k-1}\right)\approx f_{k-1}^{j}\left({\hat{X}}_{k-1}^{j},\Phi_{k-1}\right)+\frac{\partial f_{k-1}^{j}\left({\hat{X}}_{k-1}^{j},\Phi_{k-1}\right)}{\partial X_{k-1}^{j}}{|}_{X_{k-1}^{j}={\hat{X}}_{k-1}^{j}}\left(X_{k-1}^{j}-{\hat{X}}_{k-1}^{j}\right)\\ h_{k}^{j}\left(X_{k}^{j},\Phi_{k}\right)\approx h_{k}^{j}\left({\hat{X}}_{k}^{j},\Phi_{k}\right)+\frac{\partial h_{k}^{j}\left({\hat{X}}_{k}^{j},\Phi_{k}\right)}{\partial X_{k}^{j}}{|}_{X_{k-1}^{j}={\hat{X}}_{k-1}^{j}}\left(X_{k-1}^{j}-{\hat{X}}_{k-1}^{j}\right) \end{array}\right. \end{array}$$ (22)

The above expression was simplified to:

$$A_{k}^{j}=\frac{\partial f_{k}^{j}\left({\hat{X}}_{k}^{j},\Phi_{k}\right)}{\partial X_{k}^{j}}{|}_{X_{k}^{j}={\hat{X}}_{k}^{j}},\;C_{k}^{j}=\frac{\partial h_{k}^{j}\left({\hat{X}}_{k}^{j},\Phi_{k}\right)}{\partial X_{k}^{j}}{|}_{X_{k-1}^{j}={\hat{X}}_{k-1}^{j}}$$

Eq (12) can then be expressed as:

$$\begin{array}{c} \left\{ \begin{array}{c} X_{k}^{j}=A_{k}^{j}X_{k-1}^{j}+f_{k-1}^{j}\left({\hat{X}}_{k-1}^{j},\Phi_{k-1}\right)-A_{k}^{j}{\hat{X}}_{k-1}^{j}+w_{k-1}^{j}\\ Z_{k}=C_{k}^{j}X_{k}^{j}+h_{k}^{j}\left({\hat{X}}_{k}^{j},\Phi_{k}\right)-C_{k}^{j}{\hat{X}}_{k}^{j}+v_{k} \end{array}\right. \end{array}$$ (23)

For the nonlinear system given by Eq (12), the designed STKF was

$$\begin{array}{c} {\hat{X}}_{k+1}^{j}={\hat{X}}_{k}^{j}+L_{k}^{j}r_{k}^{j} \end{array}$$ (24)

$$\begin{array}{c} r_{k}^{j}=Z_{k}-h_{k}^{j}\left({\hat{X}}_{k}^{j},\Phi_{k}\right) \end{array}$$ (25)

In this study, $r_{k}^{j}$ is the sensor current residuals of SRM power converter under the *j* model. Here, the sensor current residuals of the Eq (19) and the observation coefficients of each fault model can be used to calculate the model probability. IMM adopted the method based on model probability for fault detection and fault separation, which is introduced in the third step of “Model probability calculation”.

The strong tracking property of the filter was to determine a gain matrix $L_{k}^{j}$ online. $L_{k}^{j}$ must satisfy the following conditions:

$$\begin{array}{c} \left\{ \begin{array}{c} E\left[\left(X_{k}^{j}-{\hat{X}}_{k}^{j}\right)\right]\left[{\left(X_{k}^{j}-{\hat{X}}_{k}^{j}\right)}^{T}\right]=\text{min}\\ E[r_{k}^{j}{\left(r_{k}^{j}\right)}^{T}]=0 \end{array}\right. \end{array}$$ (26)

Eq (20) indicated that when there was a great difference between the state estimate and the actual value, $L_{k}^{j}$ was determined to be online.The residual was orthogonal to keep an accurate track of the system. If the system model was accurate, then STKF dearaded to EKF. By doing so, the amount of computation of STKF was also moderate.

The error covariance matrix was:

$$\begin{array}{c} P_{k}^{j}=\lambda_{k}^{j}A_{k-1}^{j}P_{k-1}^{j}A_{k-1}^{j}+Q_{k-1}^{j} \end{array}$$ (27)

$\lambda_{k}^{j}$ was fading factor, and $\lambda_{k}^{j}\geq 1$.

The fading factor was solved:

$$\begin{array}{c} \lambda_{k}^{j}=\{\begin{array}{cc} e_{k}^{j}, & e_{k}^{j}£1\\ 1, & e_{k}^{j}\leq 1 \end{array} \end{array}$$ (28)

$$e_{k}^{j}=tr\left(N_{k}^{j}\right)/tr\left(M_{k}^{j}\right).$$

Where $N_{k}^{j}$ and $M_{k}^{j}$ were defined as:

$$\begin{array}{c} \left\{ \begin{array}{c} N_{k}^{j}=E_{0,k}^{j}-\beta^{j}R_{k}-C_{k}^{j}Q_{k-1}^{j}{\left(C_{k}^{j}\right)}^{T}\\ M_{k}^{j}=C_{k}^{j}A_{k-1}^{j}P_{k-1}^{j}{\left(A_{k-1}^{j}\right)}^{T}{\left(C_{k}^{j}\right)}^{T} \end{array}\right. \end{array}$$ (29)

Where $E_{0,k}^{j}$ was the residual covariance matrix, and its calculation formula is:

$$\begin{array}{c} E_{0,k}^{j}=\{\begin{array}{c} r_{1}^{j}{\left(r_{1}^{j}\right)}^{T}\left(k=1\right)\\ \frac{\rho E_{0,k-1}^{j}+r_{k}^{j}{\left(r_{k}^{j}\right)}^{T}}{1+\rho }\left(k>1\right) \end{array} \end{array}$$ (30)

*ρ* was the forgetting factor, *β* was the weakening factor, and *β* ≥ 1.

Then, the Kalman gain matrix was:

$$\begin{array}{c} L_{k}^{j}=P_{k}^{j}{\left(C_{k}^{j}\right)}^{T}{\left[C_{k}^{j}P_{k}^{j}{\left(C_{k}^{j}\right)}^{T}+R_{k}\right]}^{-1} \end{array}$$ (31)

The error covariance update matrix was:

$$\begin{array}{c} P_{k}^{j}=\left(I-L_{k}C_{k}\right)P_{k-1}^{j} \end{array}$$ (32)

Where *I* was dentity matrix.

3. Model probability calculation

Fault detection and fault isolation were determined by the matching model, and the threshold value of the model probability is set as *μ*_*T*_ ∈ (0.5, 1)]. The model probability $\mu_{k}^{j}$ was updated by the likelihood function method, and likelihood function of *m*_*j*_ model was:

$$\begin{array}{c} \Lambda_{k}^{j}=\frac{e^{-\frac{1}{2}{\left(r_{k}^{j}\right)}^{T}r_{k}^{j}{\left(S_{k}^{j}\right)}^{-1}}}{2\pi^{n/2}|S_{k}^{j}{|}^{1/2}} \end{array}$$ (33)

And

$$\begin{array}{c} \left\{ \begin{array}{c} S_{k}^{j}=C_{k}^{j}P_{k|k-1}^{j}{\left(C_{k}^{j}\right)}^{T}+Q_{k}^{j}\\ r_{k}^{j}=Z_{k}-h_{k}^{j}\left({\hat{X}}_{k}^{j},\Phi_{k}\right) \end{array}\right. \end{array}$$ (34)

Then probability of *m*_*j*_ model was:

$$\begin{array}{c} \mu_{k}^{j}=\frac{\Lambda_{k}^{j}{\bar{c}}_{j}}{\sum_{j=1}^{r}\Lambda_{k}^{j}{\bar{c}}_{j}} \end{array}$$ (35)

For model *m*_*j*_, if the following formula was satisfied:

$$\begin{array}{c} \mu_{k}^{j}=\text{max}\{\mu_{k}^{j}{|}_{i=0}^{M}\}>\mu_{T} \end{array}$$ (36)

And $\mu_{k-1}^{j}>\mu_{T}$, it was considered that *j* fault had occurred, that is, the *j* fault had been detected.

Combining (16) and (29), the model probability $\mu_{k}^{j}$ was observed to determine the matching model of the current system. Meanwhile, the mixed probability $\mu_{k-1|k-1}^{ij}$ determined the mixing of input information, consequently affecting the residual $r_{k}^{j}$. $\mu_{k}^{j}$ and $\mu_{k-1|k-1}^{ij}$ were determined by transition probability matrix, so transition probability matrix determined the information interaction and model transformation among the fault models in IMM.

4. Fused output

Based on the model probability, the weighted combined value of each filter was estimate:

Total state estimate:

$$\begin{array}{c} {\hat{X}}_{k|k}=\sum_{j=1}^{l}{\hat{X}}_{k|k}^{j}\mu_{k}^{j} \end{array}$$ (37)

Total variance of state estimate:

$$\begin{array}{c} P_{k|k}=\sum_{j=1}^{l}\mu_{k}^{j}\left[P_{k|k}^{j}+\left({\hat{X}}_{k|k}^{j}-{\hat{X}}_{k|k}\right){\left({\hat{X}}_{k|k}^{j}-{\hat{X}}_{k|k}\right)}^{T}\right] \end{array}$$ (38)

#### Noise suppression algorithm in the non-moel transformation stage

When there was no model switching in the system, the main error came from noise in the current model information. The his torical model information contained the matched model information that was less affected by noise. If this information can be used to correct the transition probability, raise the probability that other models transitioned to the matched model and lower the probability that the matched model transitioned to other models, then the effect of noise on the proportion of the matched model can be effectively suppressed [45]. Accordingly, it was evident that if a transition probability correction function was constructed using the historical model information, then an adequate noise reduction effect can be achieved. For the IMM algorithm, after iteration, the model probabilities *μ* would be produced. Generally speaking, these model probabilities were only used for the fused output of information without being fully utilized by the algorithm. For the iteration at time *k*, the model probability at time *k* − *N* contained the historical information of model probability. The change rate of model probability *μ*_*k*−*N*_ can be used to effectively express the variation trend of probability. The difference equation of the n-th order consequent of the model probability of *m*_*j*_ was:

$$\begin{array}{c} \Delta \mu_{k}^{j}\left(N\right)=\mu_{k}^{j}-\mu_{k-N}^{j},\;\;1\leq N\leq k \end{array}$$ (39)

The transition probability correction function of the model *m*_*j*_ built with the above formula was:

$$\begin{array}{c} f_{j}\left(k\right)=\frac{1}{1-\Delta \mu_{k}^{j}\left(N\right)},j=1,2,3,\ldots ,l \end{array}$$ (40)

The transition probability correction function of the model *m*_*j*_*f*_*j*_(*k*), was used to correct the transition probability that other models transitioned to this model:

$$\begin{array}{c} {\left(\pi_{k}^{ij}\right)}^{\prime }=f_{j}\left(k\right)*\pi_{k-1}^{ij},i=1,2,3,\ldots ,l \end{array}$$ (41)

As seen in (13), the corrected transition probability must be normalized:

$$\begin{array}{c} {\left(\pi_{k}^{ij}\right)}^{1}=\frac{{\left(\pi_{k}^{ij}\right)}^{\prime }}{\sum_{j=1}^{m}{\left(\pi_{k}^{ij}\right)}^{\prime }} \end{array}$$ (42)

Using the above method, the transition probability correction functions of all models were calculated, and the transition probabilities were corrected. Notably, when the model probability rose, $\Delta \mu_{k}^{j}\left(N\right)>0$, suggesting that the real model tended to transition to the model *m*_*j*_. At this point, the transition probability correction function of this model was *f*_*j*_(*k*) > 1. The transition probability *π*^*ij*^ was then corrected and normalized with *f*_*j*_(*k*). Hence, the corrected transition probability matrix was ${\left(\pi_{k}^{ij}\right)}^{1}>{\left(\pi_{k-1}^{ij}\right)}^{1}$. Here, there existed a model *m*_*i*_, whose $\Delta \mu_{k}^{i}\left(N\right)<0$, then *f*_*i*_(*k*) < 1. Therefore, after correction, ${\left(\pi_{k}^{ji}\right)}^{1}<{\left(\pi_{k-1}^{ji}\right)}^{1}$. Apparently, these two changes were very benefificial in increasing the proportion of the probability of the model *m*_*j*_.

#### Delay reduction algorithm in the model transformation stage

In the model switching stage, the main goal in correcting the model probability was to ensure it transformed quickly when the system mode changed [46]. The likelihood function of the model can also mirror the matching degree between the model and system. The greater the likelihood function, the higher the matching degree. In addition, when the model changed, corresponding changes would occur as well. If such information may be used rationally, then we can effectively judge whether there was model transformation in the current system. Therefore, the likelihood function ratio between the current matched model *m*_*e*_ and other models *m*_*i*_ was defifined as:

$$\begin{array}{c} \Lambda_{ei}=\frac{\Lambda_{e}}{\Lambda_{i}}e=1,2,3,\ldots l,\;\text{and}\;e\neq i \end{array}$$ (43)

When there was no model transformation in the system, *m*_*e*_ was the real model of the system, and its likelihood function was greater than that of other models. The model jump threshold was set to *Th*. Under this circumstance, min(Λ_*ei*_) > *Th*. Moreover, when there was model transformation in the system, its likelihood function was smaller than that of other models, that is, min(Λ_*ei*_) < *Th*. In other words, whether there was transformation in the system can be judged by the relationship between min(Λ_*ei*_) and the model jump threshold *Th*.

A transition probability correction function was then reconstructed using the current information of the system obtained according to the above method. Here, the transition probability correction function of the model *m*_*j*_ at time *k* was:

$$\begin{array}{c} f_{j}^{\prime }\left(k\right)=\frac{1}{1-[\left(1-a\right)\left(\mu_{k}^{j(A)}-\mu_{k-1}^{j(A)}\right)+a\left(\mu_{j}^{k(C)}-\mu_{j}^{k(C)}\right)]},j=1,2,3,\ldots ,l \end{array}$$ (44)

$$\begin{array}{c} a=\{\begin{array}{cc} 1, & \text{min}(\Lambda_{ei})<Th\\ 0, & \text{min}(\Lambda_{ei})>Th \end{array} \end{array}$$ (45)

where $\mu_{j}^{k(C)}$ represented the probability of the model *m*_*j*_ in the standard the IMM algorithm, and $\mu_{k}^{j(A)}$ represented the probability that the historical information contained in the model transitioned to the model *m*_*j*_ in the adaptive the IMM algorithm. *a* was the model transformation coeffificient, which was used to introduce the current model information to the transition probability correction function. When *a* was 0, then no model transformation took place. In this case, (37) degraded to (33). Furthermore, when *a* was 1, model transformation was considered to occur. The transition probability correction function can simply be defifined by the change rate of model probability $\mu_{j}^{k(C)}$ that contained a small amount of historical model information. In this case, the transition probability correction function was:

$$\begin{array}{c} {\left(\pi_{k}^{ij}\right)}^{\prime \prime }=f_{j}^{\prime }\left(k\right)\times \pi_{k-1}^{ij},i=1,2,3,\ldots ,l \end{array}$$ (46)

$$\begin{array}{c} {\left(\pi_{k}^{ij}\right)}^{2}=\frac{{\left(\pi_{k}^{ij}\right)}^{\prime \prime }}{\sum_{j=1}^{l}{\left(\pi_{k}^{ij}\right)}^{\prime \prime }} \end{array}$$ (47)

In light of the above conclusions, the model jump threshold *Th* determined the timing of introducing different delayed information. Moreover, the value of *Th* had a great influence on the performance of the algorithm. In a particular study [47], the principle of obtaining the *Th* value was outlined by conducting a systematic experiment. This paper analyzed the value of *Th* based on that study and combined it with the specifific characteristics of SRM power converter, which is summarized below:

1. In terms of SRM power converter, there are three equivalent circuit models under normal operation, and even if the system is not converted to a fault model, it will frequently be converted between the three equivalent circuit models. The model jump in the main state. Therefore, the model switching threshold should not be too small, and the value range of *Th* should be set at (0.5, 1];
2. In the set value range, the larger the *Th*, the faster the SRM power converter model conversion speed, but the worse the noise suppression ability. Moreover, the smaller the *Th*, the slower the SRM power converter model conversion speed, but the stronger the noise suppression ability;
3. The value of *Th* should be directly related to the measurement noise of the SRM power converter model. In this paper, through experiments with different the value of *Th*, it was found that when the value range of *Th* was within the range [0.7, 0.8], conversion speed of the SRM power converter system model and the noise suppression capability were optimal;
4. In order to balance the noise suppression capability and conversion speed of the model, this paper took the average value of [0.7, 0.8], that is, *Th* = 0.75

### Realization of multi-fault diagnosis algorithm for SRM power converter

#### Establishment of the fault model set

In this paper, the normal working model, open circuit and short circuit fault model of each switch tube in the three phases A, B and C were established for the fault of each switch tube in the SRM power converter.

In section of the establishing an equivalent model for the SRM power converter, considering the freewheeling bus current in SRM power converter, it can be expressed as:

$$\begin{array}{c} i_{fup}=\bar{S_{A2}}i_{a}+\bar{S_{B2}}i_{b}+\bar{S_{C2}}i_{c} \end{array}$$ (48)

$$\begin{array}{c} i_{fdn}=\bar{S_{A1}}i_{a}+\bar{S_{B1}}i_{b}+\bar{S_{C1}}i_{c} \end{array}$$ (49)

Substituting Eqs (42) and (49) into Eqs (4)–(6):

$$\begin{array}{c} \left\{ \begin{array}{c} i_{1}=\left(m_{1}+n\right)i_{a}+m_{2}i_{b}+m_{3}i_{c}\\ i_{2}=m_{1}i_{a}+\left(m_{2}+n\right)i_{b}+m_{3}i_{c}\\ i_{3}=m_{1}i_{a}+m_{2}i_{b}+\left(m_{3}+n\right)i_{c} \end{array}\right. \end{array}$$ (50)

Among them:

$$\begin{array}{c} \left\{ \begin{array}{c} m_{1}=2\bar{S_{A1}}-\bar{S_{A2}}\\ m_{2}=2\bar{S_{B1}}-\bar{S_{B2}}\\ m_{3}=2\bar{S_{C1}}-\bar{S_{C2}} \end{array}\right. \end{array}$$ (51)

According to the above formula, *m*_1_, *m*_2_ and *m*_3_ are completely determined by the on-off state of the switch tube and can only take values from the four numbers of 0, ±1 and 2. The specific corresponding relationship is shown in Table 1.

**Table 1:** Relationship between the on-off state of the switch tube and *m*_1_, *m*_2_, *m*_3_.

SA1	SA2	*m*_1_	SB1	SB2	*m*_2_	SC1	SC2	*m*_3_
0	0	1	0	0	1	0	0	1
0	1	2	0	1	2	0	1	2
1	0	−1	1	0	−1	1	0	−1
1	1	0	1	1	0	1	1	0

Rewriting formula (50) in matrix form:

$$\begin{array}{c} \left[\begin{array}{c} i_{1}\\ i_{2}\\ i_{3} \end{array}]=[\begin{array}{ccc} m_{1}+n & m_{2} & m_{3}\\ m_{1} & m_{2}+n & m_{3}\\ m_{1} & m_{2} & m_{3}+n \end{array}][\begin{array}{c} i_{a}\\ i_{b}\\ i_{c} \end{array}\right] \end{array}$$ (52)

Regardless of the working state of the motor, if the phase current is calculated correctly, it is necessary to ensure that there is a unique solution in Eq (52). Therefore, the observation coefficient matrix must satisfy:

$$\begin{array}{c} rank[\begin{array}{ccc} m_{1}+n & m_{2} & m_{3}\\ m_{1} & m_{2}+n & m_{3}\\ m_{1} & m_{2} & m_{3}+n \end{array}]=3 \end{array}$$ (53)

Here, *rank*[] indicated the rank of the matrix;

The simulation results showed that when *n* = 1, the formula (53) was guaranteed to be true in any state.

By discretizing Eqs (14) and (52), the system state equation and measurement equation corresponding to Eq (15) was obtained as follows:

$$\begin{array}{c} \begin{array}{cc} \left[\begin{array}{c} i_{a,k}\\ i_{b,k}\\ i_{c,k} \end{array}\right] & =[\begin{array}{ccc} e^{-\frac{T}{\tau_{1}}} & 0 & 0\\ 0 & e^{-\frac{T}{\tau_{2}}} & 0\\ 0 & 0 & e^{-\frac{T}{\tau_{3}}} \end{array}][\begin{array}{c} i_{a,k-1}\\ i_{b,k-1}\\ i_{c,k-1} \end{array}]\\ & +[\begin{array}{ccc} \frac{1-e^{-\frac{T}{\tau_{1}}}}{R_{a}} & 0 & 0\\ 0 & \frac{1-e^{-\frac{T}{\tau_{2}}}}{R_{b}} & 0\\ 0 & 0 & \frac{1-e^{-\frac{T}{\tau_{3}}}}{R_{c}} \end{array}][\begin{array}{c} U_{La,k}\\ U_{Lb,k}\\ U_{Lc,k} \end{array}]+[\begin{array}{c} \omega_{1,k}\\ \omega_{2,k}\\ \omega_{3,k} \end{array}] \end{array} \end{array}$$ (54)

$$\begin{array}{c} \left[\begin{array}{c} i_{1,k}\\ i_{2,k}\\ i_{3,k} \end{array}]=[\begin{array}{ccc} m_{1}+1 & m_{2} & m_{3}\\ m_{1} & m_{2}+1 & m_{3}\\ m_{1} & m_{2} & m_{3}+1 \end{array}][\begin{array}{c} i_{a,k}\\ i_{b,k}\\ i_{c,k} \end{array}]+[\begin{array}{c} v_{1,k}\\ v_{2,k}\\ v_{3,k} \end{array}\right] \end{array}$$ (55)

The state variable was $X_{k}=[\begin{array}{ccc} i_{a,k} & i_{b,k} & i_{c,k} \end{array}{]}^{T}$, the control variable was $U_{L,k}=[\begin{array}{ccc} U_{La,k} & U_{Lb,k} & U_{Lc,k} \end{array}{]}^{T}$ and the observation variable was $Z_{k}=[\begin{array}{ccc} i_{1} & i_{2} & i_{3} \end{array}{]}^{T}$.

Comparison formula (15), at this time, each coefficient matrix of nonlinear equations were determined:

$$\begin{array}{cc} & A_{k-1}=[\begin{array}{ccc} e^{-\frac{T}{\tau_{1}}} & 0 & 0\\ 0 & e^{-\frac{T}{\tau_{2}}} & 0\\ 0 & 0 & e^{-\frac{T}{\tau_{3}}} \end{array}],B_{k-1}=[\begin{array}{ccc} \frac{1-e^{-\frac{T}{\tau_{1}}}}{R_{a}} & 0 & 0\\ 0 & \frac{1-e^{-\frac{T}{\tau_{2}}}}{R_{b}} & 0\\ 0 & 0 & \frac{1-e^{-\frac{T}{\tau_{3}}}}{R_{c}} \end{array}],\\ & C_{k}=[\begin{array}{ccc} m_{1}+1 & m_{2} & m_{3}\\ m_{1} & m_{2}+1 & m_{3}\\ m_{1} & m_{2} & m_{3}+1 \end{array}] \end{array}$$

When any switch tube in the power converter is in the normal, open and short circuit state, the corresponding observation coefficients (*m*_1_, *m*_2_ and *m*_3_) can exist, and the observation coefficient matrix *C*_*k*_ changes accordingly. Taking phase A as an example, Table 2 shows the corresponding relationship between the switching tube state and *m*_1_, *m*_2_ and *m*_3_. Evidently, three models are under normal conditions: open circuit SA1, open circuit SA2 and open circuit SA1 and SA2, respectively, which correspond to one model. Meanwhile, short circuit SA1, short circuit SA2 and short circuit SA1 and SA2, respectively, correspond to two models.

**Table 2:** Corresponding relationship between state of phase A switch tube and *m*_1_, *m*_2_ and *m*_3_.

Tube state	Ta	Tb	Tc	*m*_1_	*m*_2_	*m*_3_
**Normal**	1	−1	−1	0	1	1
	−1	1	−1	1	0	1
	−1	−1	1	1	1	0
**SA1 open circuit**	0	−1	−1	2	1	1
**SA2 open circuit**	0	−1	−1	−1	1	1
**SA1, SA2 open circuit**	−1	−1	−1	1	1	1
**SA1 short circuit**	0	1	−1	−1	0	1
	0	−1	1	−1	1	0
**SA2 short circuit**	0	1	−1	2	0	1
	0	−1	1	2	1	0
**SA1, SA2 short circuit**	1	1	−1	0	0	1
	1	−1	1	0	1	0

Assuming that there are altogether *j* models of normal, open circuit and short circuit of each switch tube in the power converter circuit, the estimated ${\hat{Z}}_{k}^{j}$ of sensor current under the *j* model is a function of state variables *X*_*k*_ and control variables *U*_*L*,*k*_, and the estimated ${\hat{Z}}_{k}^{j}$ of sensor current can be sorted into the following functions:

$$\begin{array}{c} {\hat{Z}}_{k}^{j}=g\left({\hat{X}}_{k}^{j},U_{L,k}\right) \end{array}$$ (56)

The terminal voltage residual of the model can be expressed as

$$\begin{array}{c} r_{k}^{j}=Z_{k}^{j}-{\hat{Z}}_{k}^{j} \end{array}$$ (57)

When designing the the transition probability matrix, it is appropriate to correspond diagonal elements (that is, the transition probability of each model to itself) to the average residence time of each mode. That is $\pi_{ii}=\text{max}\{l_{i},\frac{1-T}{\tau_{i}}\}$, where *T* is the sampling period of the system, the expected residence time of the *i* model of the system is *τ*_*i*_, and the lower limit designed for the transition probability of the *i* model is *l*_*i*_.

After the diagonal elements of the transition probability matrix were determined, the transition probability from normal model to fault model was immediately determined. Without considering the causality of the fault models in the model set, these faults do not occur one after another, hence, the conversion from the fault model to another fault model can be ignored.

#### Multi-fault diagnostic process

**Fig 6:**
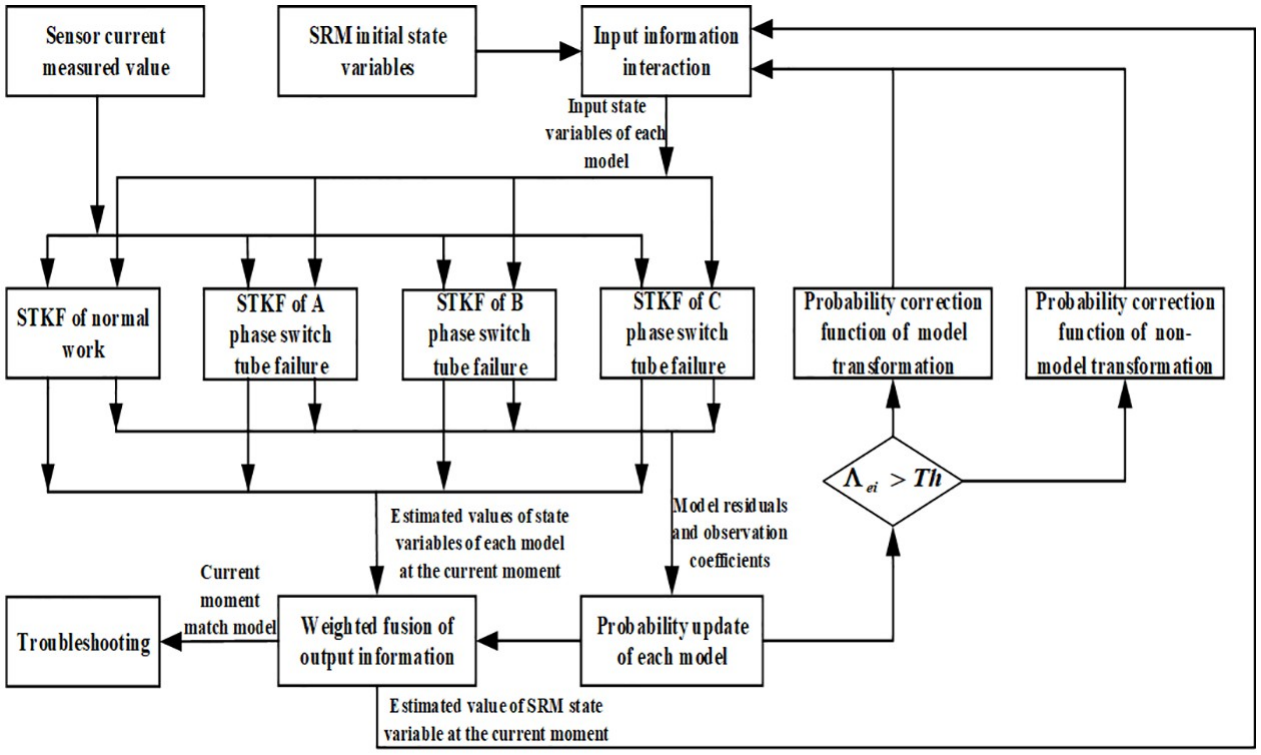
Flow chart of multi-fault diagnosis of SRM power converter based on IMM.

In light of the above data, the model equation was initialized. The initialization parameters included the transition probability matrix, probability matrix of the model, coefficients A, B and C of the nonlinear equation, covariance matrix *P* and jump threshold *Th*, which were substituted into the fault diagnosis algorithm of IMM for operation. The detailed flow chart of multi-fault diagnosis is given in Fig 6.

## Simulation analysis

**Fig 7:**
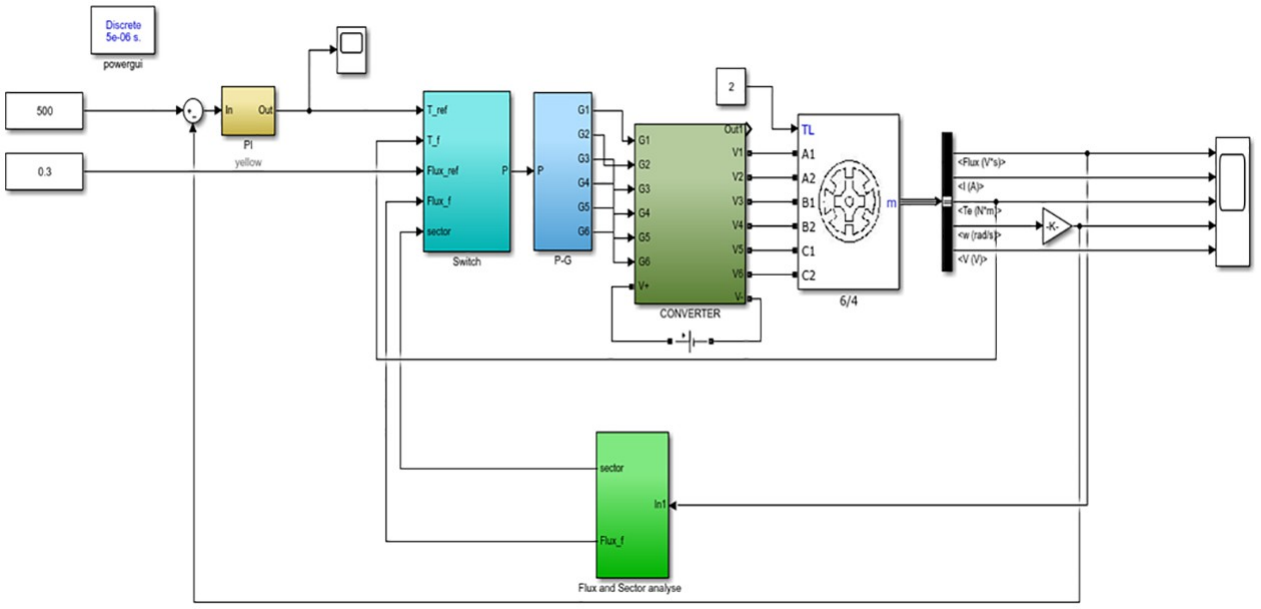
Simulation model of 6/4 pole SRM system simulation mode.

This paper adopted the 6/4-pole SRM system simulation model, which was mainly divided into the SRM ontology model, power converter module, voltage vector switch table module (including Switch and P-G), and flux change and sector judgment module, as shown in Fig 7. The outer ring was a speed ring, while the output was a given value of torque through PI adjustment. Meanwhile, the inner loop represented torque and flux linkage. According to the difference between the feedback value and given value, the change requirements were given. Combined with the position of flux linkage, the voltage vector was selected to control the on and off of the SRM phase winding. The simulation parameters were then set according to the following: winding internal resistance of 0.15Ω, moment of inertia of 0.0082 kg ⋅ m^2^, damping coefficient of 0.008 N ⋅ m ⋅ s, load torque of 10 N ⋅ m, DC power supply of 310 V, given flux linkage of 0.3 Wb, rated speed of 3000 r/min, and flux linkage and torque hysteresis width of 0.1 Wb and 0.1 N ⋅ m, respectively. The gate switch control signal of the power switch device was then set, and the corresponding three-phase control vector action sequence was *A* − *C* − *B* − *A*. The occurrence of short-circuit and open-circuit faults of switching tubes was simulated by switching the switching tubes both in parallel and in series in the power converter module.

Equivalent circuit model involved in the Simulation analysis: normal operation, multiple faults occurring in turn and multiple faults occurring simultaneously. The equivalent circuit models involved in the experiment included the normal working model, open-circuit fault model of A-phase SA1, open-circuit fault model of A-phase SA1 and short-circuit fault model of B-phase SB1, open-circuit fault model of A-phase SA2, short-circuit fault model of C-phase SC1 and short-circuit fault model of C-phase SC2. Using the IMM multi-fault diagnosis algorithm cycle discussed in the third section, multi-fault detection as well as the separation of SRM power converter faults were then carried out. The threshold *μ*_*T*_ = 0.90 was selected, after which the simulation time was 0.3 s. According to Table 2, three normal working models are evident in the order of the three-phase control vectors, namely (0, 1, 1), (1, 0, 1) and (1, 1, 0). Moreover, one open-circuit fault model (2, 1, 1) was present for SA1 in phase A, two models (2, −1, 1) and (1, −1, 0) for SB1 in phase B, and two models (−1, 1, 0) and (1, 0, 0) for SA2 in phase A, phase SC1 of C and SC2 in phase C. According to the above eight state models, the corresponding STKF was set, with model 1 as (0, 1, 1), model 2 as (1, 1, 0), model 3 as (1, 0, 1), model 4 as (2, 1, 1), model 5 as (2, −1, 1), model 6 as (1, −1, 0), model 7 as (−1, 1, 0) and model 8 as (1, 1, 0). Furthermore, the SRM power converter multi-model simulation system was composed of each of them. The initial probability values of all models were 1/8.

### SRD normal operation simulation

#### SRD simulation under different loads

**Fig 8:**
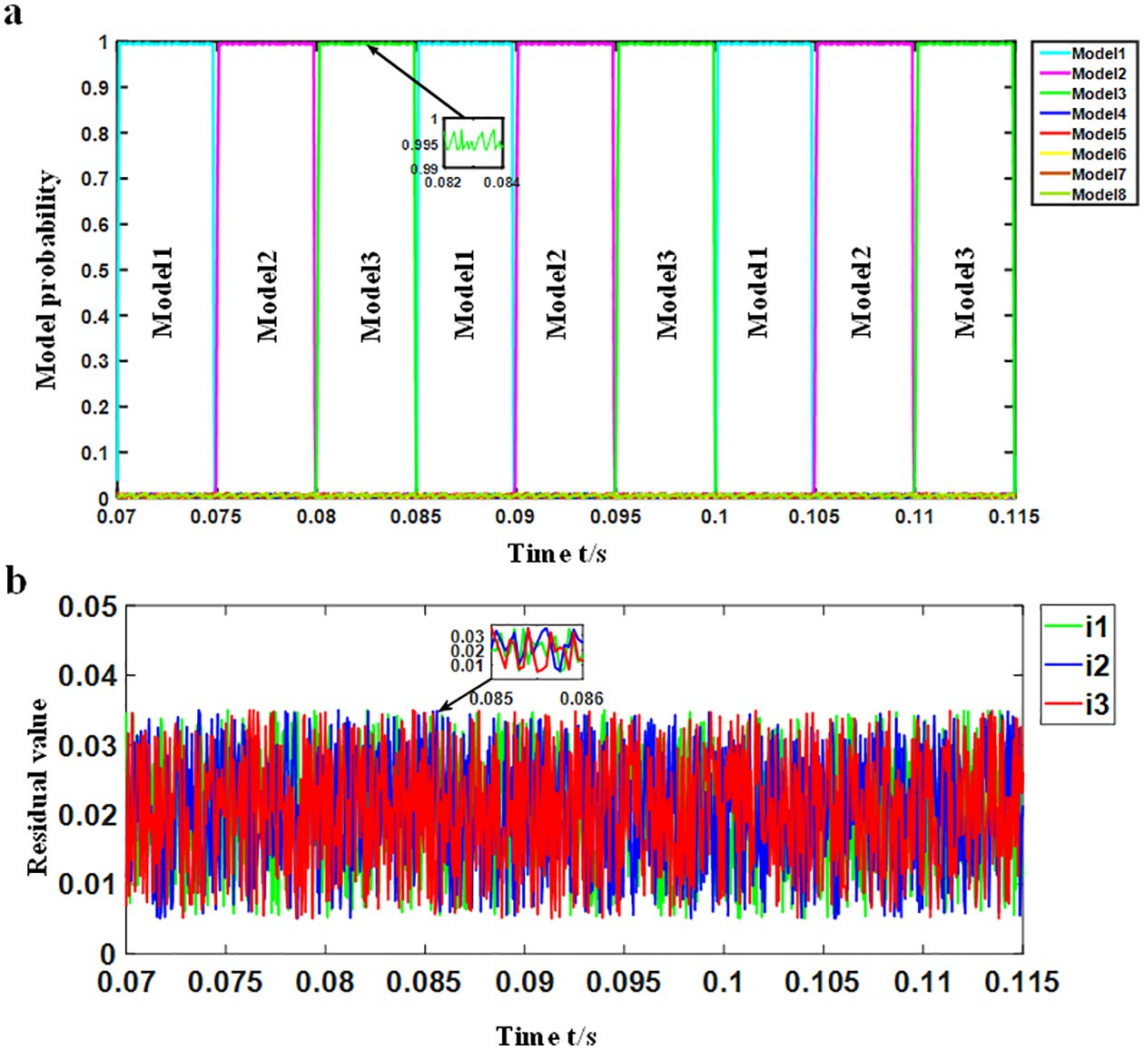
Simulation waveform under rated load. (a) Probability change waveform of each model (b) Current residual waveform of each.

Experiment: The motor ran at the rated speed, and the load was set to no-load, half-load and rated load, respectively. The simulation waveform under the rated load is shown in Fig 8.

As shown in Fig 8a, in each time period, the probability of a single model was close to 1, that is, the matching model. Meanwhile, the probability of the matching model is shown in Table 3, for which the probability of the other models was found to be close to 0. When no fault was detected, models 1, 2 and 3 were alternately matched with the current system, that is, the system was in the normal working mode, and no time delay was present when switching models. According to Fig 8b, the residuals of the three current sensors were all very small and remained between 0.005 and 0.035, while the residual waveforms did not greatly fluctuate when the model was switched. Similarly, the motor was placed in the no-load and half-load operation states, respectively, in which the simulation waveform was consistent with that under the rated load. The simulation results for the different loads are shown in Tables 3 and 4.

**Table 3:** Probability comparison of matching models under different loads.

Probability	Without load	Half load	Rated load
**Model 1(%)**	99.5	99.2	99.6
**Model 2(%)**	99.3	99.4	99.5
**Model 3(%)**	99.6	99.6	99.4

**Table 4:** Comparison of residual current of each sensor under different loads.

Load state	i1 average residual	i1 maximum residual error	i2 average residual error	i2 maximum residual error	i3 average residual	i3 maximum residual
**Without load**	0.021	0.034	0.022	0.035	0.021	0.034
**Half load**	0.022	0.035	0.022	0.035	0.021	0.034
**Rated load**	0.021	0.034	0.021	0.035	0.022	0.035

#### SRD simulation under different speeds

**Fig 9:**
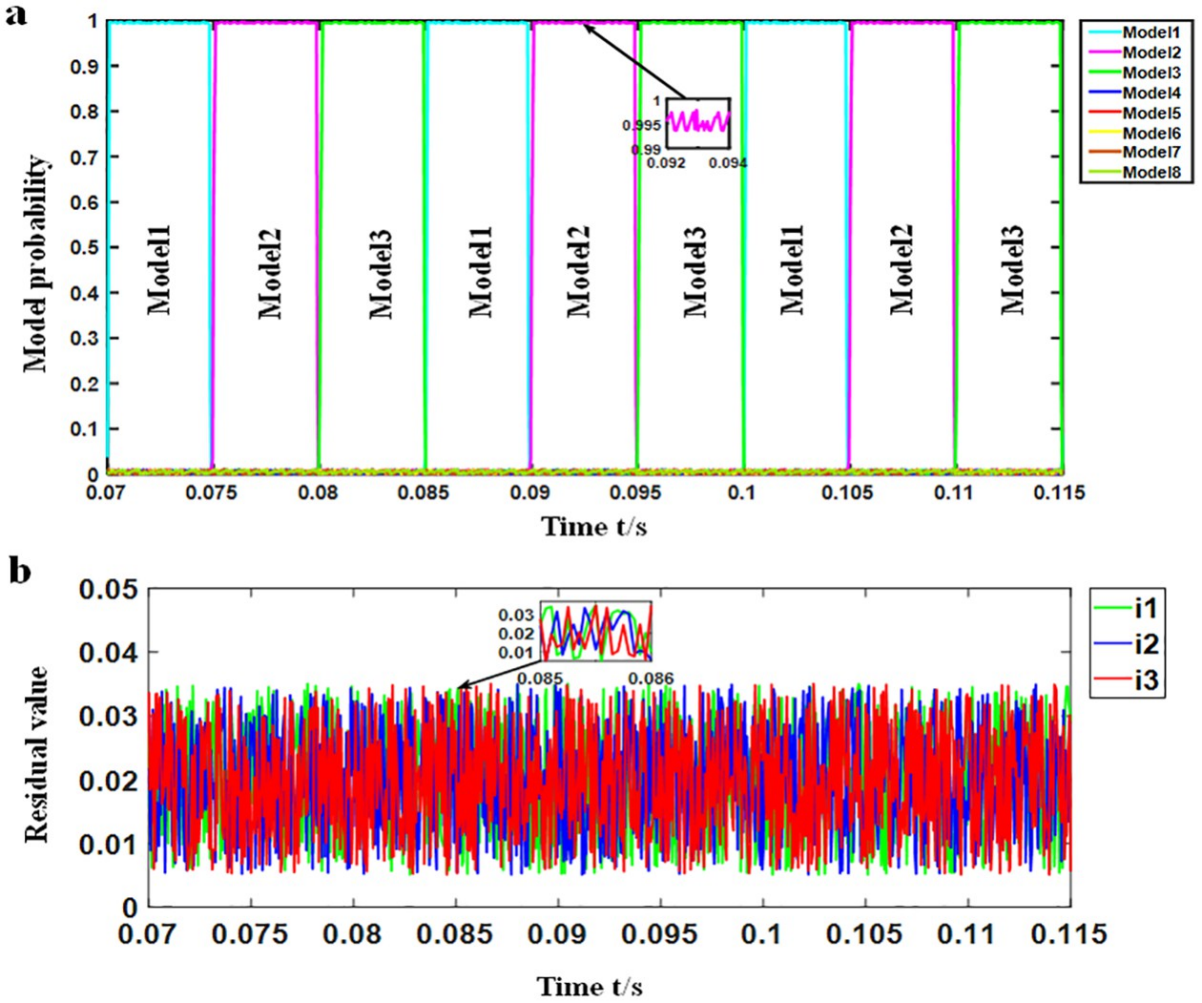
Simulation waveform at low speed. (a) Probability change waveform of each model (b) Current residual waveform of each.

Experiment: The motor was run under the rated load, and the speed was set to low speed (500 r/min), medium speed (1500 r/min) and rated speed (3000 r/min), respectively. The low-speed simulation waveform is shown in Fig 9.

According to Fig 9a, in each time period, the probability of one model was found to be close to 1, that is, the matching model. Meanwhile, the probability of the matching model is shown in Table 5, while the probability of the other models was close to 0. When no fault was detected, models 1, 2 and 3 were alternately matched with the current system, that is, the system was in the normal working mode with no time delay when switching models. As shown in Fig 9b, the current residuals of the three sensors remained between 0.005 and 0.035 like in Fig 8b, and the residual waveform did not greatly fluctuate when the model was switched. Similarly, the motor was run at medium speed and rated speed, in which the simulation waveform was found to be consistent with the simulation waveform at rated speed. The simulation results at different speeds are shown in Tables 5 and 6.

**Table 5:** Probability comparison of different speed matching models.

Probability	Low speed	Intermediate speed	Rated speed
**Model 1(%)**	99.4	99.4	99.3
**Model 2(%)**	99.5	99.5	99.5
**Model 3(%)**	99.3	99.4	99.6

**Table 6:** Comparison of residual current of each sensor at different speeds.

Speed state	i1 average residual	i1 maximum residual error	i2 average residual error	i2 maximum residual error	i3 average residual	i3 maximum residual
**Low speed**	0.021	0.034	0.022	0.035	0.021	0.034
**Intermediate speed**	0.022	0.034	0.021	0.035	0.021	0.035
**Rated speed**	0.021	0.035	0.022	0.034	0.021	0.034

The normal operation simulation results demonstrate that when there is no failure, models 1, 2 and 3 alternately match with the system and there is no failure. Meanwhile, when the SRD worked under different loads and different speeds, the probability of the matching the model reached 99%. The current residual error of each sensor was maintained at about 0.021, while the maximum residual error was 0.035. The simulation results of load mutation and speed mutation were also noted to be the same as the above results. Therefore, when the working conditions were changed, it did not affect the matching model probability as well as the current residuals of each sensor.

### SRD multi-fault simulation in turn

**Fig 10:**
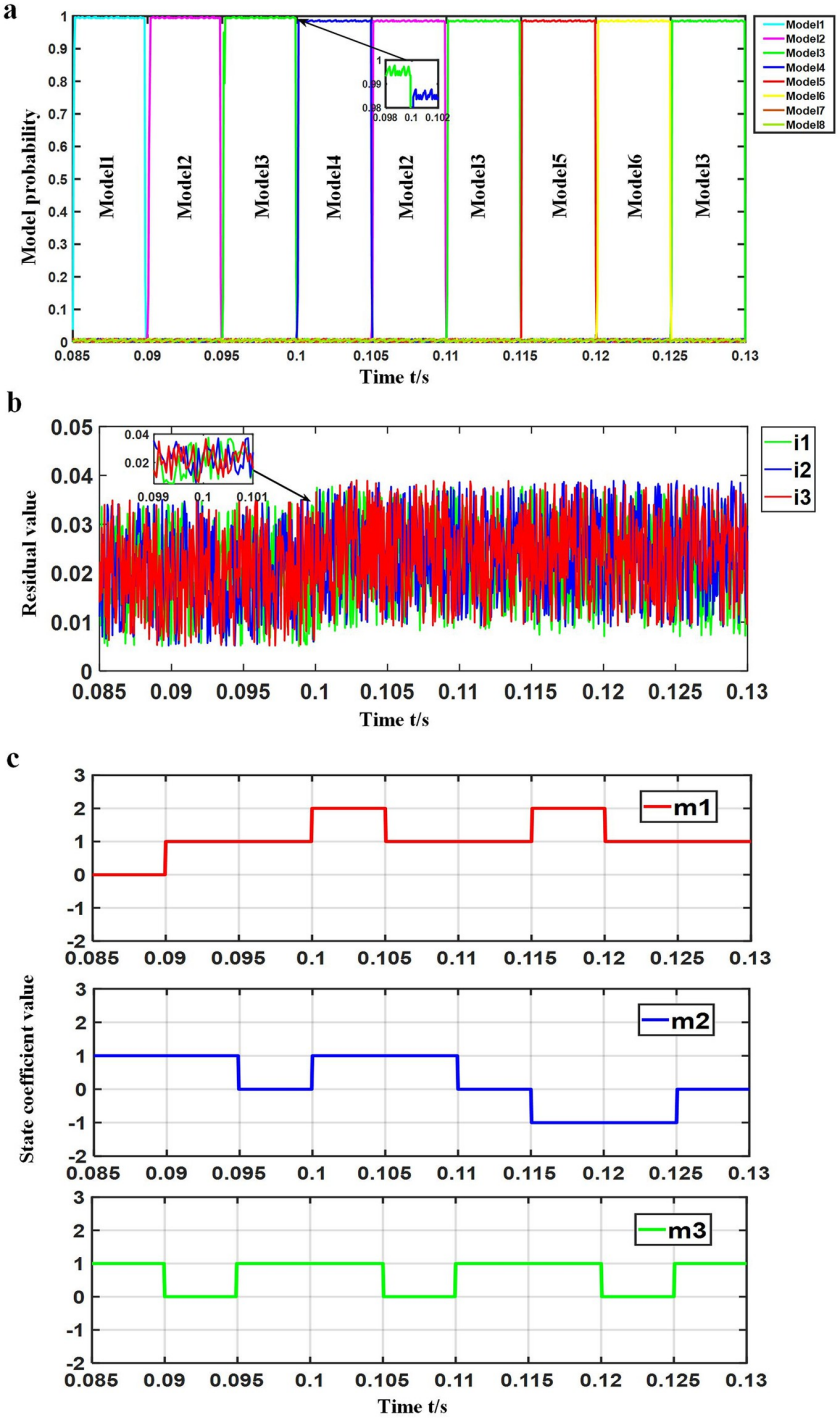
Simulation waveform of multiple faults occurring in sequence. (a) Probability change waveform of each model (b) Current residual waveform of each (c) Observation waveform of *m*_1_, *m*_2_ and *m*_3_.

Experiment: The motor was run at rated speed and rated load. At 0.1 s, the SA1 of A-phase was open-circuited, and the SB1 of B-phase was short-circuited at 0.115 s. The simulation waveform of multiple faults occurring in turn is shown in Fig 10.

As evident in Fig 10a, models 1, 2 and 3 were alternately matched with the system before 0.1 s, that is, the system was in normal working mode. Then, 0.1 s later, model 4 replaced model 1, and the probability of model 4 rapidly rose to the threshold, indicating that the current system was working in the corresponding fault mode, that is, the open circuit fault of SA1 of phase A occurred. Moreover, 0.115 s later, model 5 replaced model 4, model 6 replaced model 2, and the probability of model 5 rapidly rose to the threshold value, indicating that the current system was working in the corresponding fault mode, that is, the open-circuit fault of SA1 in phase A and the short-circuit fault of SB1 in phase B occurred simultaneously. However, the model matching probability decreased after 0.1 s. According to Fig 10b, the current residuals of the three sensors increased at 0.1 s. In order to verify that the current power converter operating state was consistent with the matching model, the *m*_1_, *m*_2_ and *m*_3_ of the current power converter operating state was observed in the experiment. As shown in Fig 10c, the current power converter operating state in the simulation with multiple faults occurring in turn and waveform of *m*_1_, *m*_2_ and *m*_3_ was observed. Here, after 0.1 s, *m*_1_ jumped from 1 to 2, *m*_2_ jumped from 0 to 1, and *m*_3_ remained at 1, which corresponded to Model 4, that is, the current power converter state was an open circuit of SA1 in phase A. After 0.115 s, *m*_1_ jumped from 1 to 2, *m*_2_ jumped from 0 to-1, and *m*_3_ remained at 1, which corresponded to the fifth model, that is, the current power converter state was the simultaneous occurrence of the SA1 in the A-phase open-circuit fault and SB1 in the B-phase short-circuit fault. Therefore, the matching model of the system was shown to be consistent with the current power converter operating state.

Table 7 shows the comparison of the probability of the matching model under the simulation of multiple faults occurring in turn. Table 8 compares the residual current of each sensor under the simulation of multiple faults occurring in turn.

**Table 7:** Comparison of probability of matching models under simulation with multiple failures occurring in sequence.

Probability	0.1 s ago	0.1 s ∼ 0.115 s	0.115 s later
**Model 1(%)**	99.4	0.1	0
**Model 2(%)**	99.5	98.7	0.1
**Model 3(%)**	99.6	98.2	98.6
**Model 4(%)**	0.1	98.5	0
**Model 5(%)**	0	0.1	98.5
**Model 6(%)**	0	0	98.3

**Table 8:** Comparison of current residual values of each sensor under simulation of multiple faults occurring in turn.

Period of time	i1 average residual	i1 maximum residual error	i2 average residual error	i2 maximum residual error	i3 average residual	i3 maximum residual
**0.1 s ago**	0.021	0.034	0.022	0.034	0.021	0.034
**0.1 s ∼ 0.115 s**	0.023	0.037	0.024	0.037	0.023	0.038
**0.115 s later**	0.023	0.038	0.023	0.038	0.024	0.038

The simulation results of multiple faults occurring in sequence illustrated that when a single fault occurred, the probability of the matching model dropped to 98%, the current residual of each sensor was maintained at about 0.023, and the maximum residual was 0.038. As a result, the system was able to accurately determine the type of fault based on the matching model. When other faults subsequently occurred, the matching probability and residual current of each sensor did not change, and a corresponding matching model was present that matched the current power converter state, thus identifying the occurrence of multiple faults and separating the faults.

### Simulation of simultaneous occurrence of multiple faults in SRD

**Fig 11:**
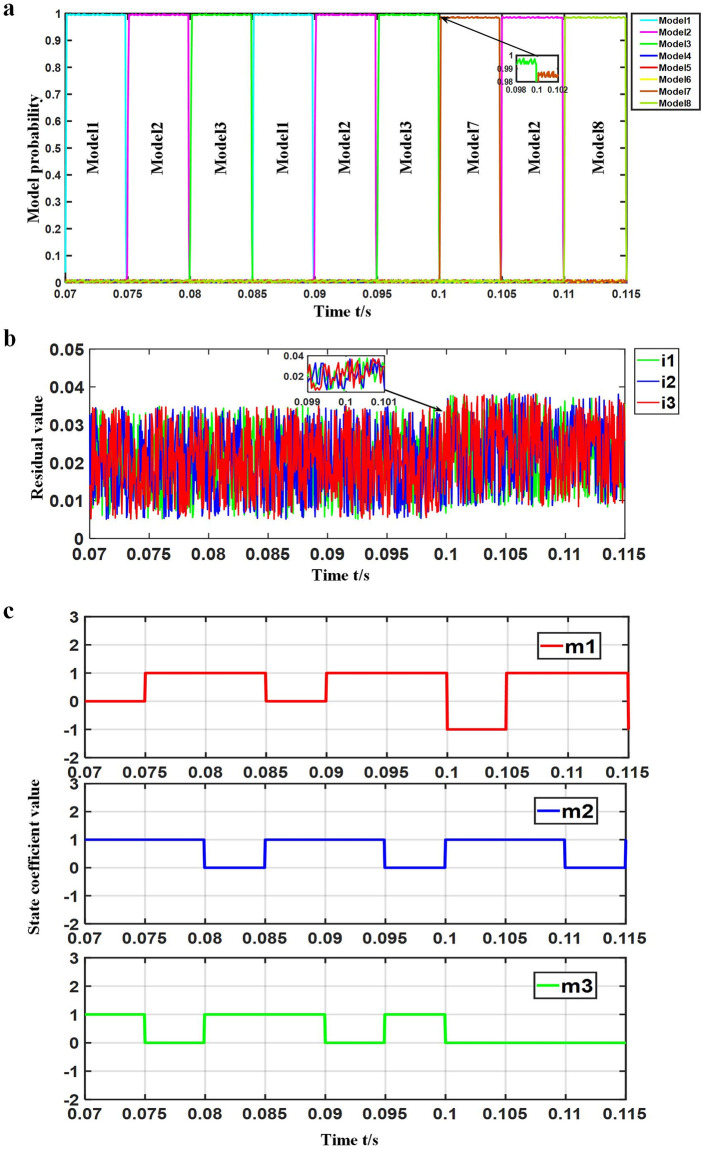
Simulation waveform of multiple faults occurring at the same time. (a) Probability change waveform of each model (b) Current residual waveform of each (c) Observation waveform of *m*_1_, *m*_2_ and *m*_3_.

Experiment setting: The motor ran at rated speed and rated load, where at 0.1 s, the short-circuit fault model of SA2 in phase A, SC1 in phase C and SC2 in phase C occurred. The simulation waveform of simultaneous occurrence of multiple faults is shown in Fig 11.

As shown in Fig 11a, the system was alternately matched with models 1, 2 and 3 before 0.1 s, that is, the system was in normal working mode. After 0.1 s, model 7 replaced model 1, model 8 replaced model 3, and the probability of model 7 rapidly rose to the threshold value, indicating that the current system was working in the corresponding fault mode, that is, the model with short-circuit fault in SA2 of phase A, short-circuit fault in SC1 of phase C and short-circuit fault in SC2 of phase C. In addition, the model matching probability was shown to not decrease. It evident in Fig 11b, the change of residual value was demonstrated to not change around 0.1 s. Fig 11c shows the current power converter running state before and after 0.1 s in the experiment of simultaneous occurrence of multiple faults, in which the waveform of *m*_1_, *m*_2_ and *m*_3_ was observed. After 0.1 s, *m*_1_ jumped from 1 to −1, *m*_2_ jumped from 0 to 1, and *m*_3_ jumped from 1 to 0, which corresponded to model 7, that is, the current power converter state was SA2 in phase A short-circuit fault, SC1 in phase C short-circuit fault and SC2 in the phase C short-circuit fault model, which was found to be consistent with the matching model of the system.

Table 9 outlines the comparison of the probability of the matching models under the simulation of simultaneous occurrence of multiple faults. Table 10 compares the residual current of each sensor under the simulation of simultaneous occurrence of multiple faults.

**Table 9:** Comparison of probability of matching models under simulation of simultaneous occurrence of multiple faults.

Probability	0.1 s ago	0.1 s later
**Model 1(%)**	99.2	0
**Model 2(%)**	99.5	0.1
**Model 3(%)**	99.6	98.4
**Model 7(%)**	0.1	98.3
**Model 8(%)**	0	98.3

**Table 10:** Comparison of residual current of each sensor under the simulation of simultaneous occurrence of multiple faults.

Period of time	i1 average residual	i1 maximum residual error	i2 average residual error	i2 maximum residual error	i3 average residual	i3 maximum residual
**0.1 s ago**	0.021	0.034	0.021	0.035	0.022	0.034
**0.1 s later**	0.024	0.038	0.025	0.037	0.024	0.038

The simultaneous multi-fault simulation findings demonstrated that when multiple faults occurred simultaneously, the matching model probability also dropped from 99% to 98%, the current residual of each sensor was maintained at about 0.023, and the maximum residual was 0.038. The system was still able to accurately match the model and determined the occurrence of multiple faults while isolating them.

### Compared with other methods

SIS and VMD-MPE are introduced for comparison in this paper to verify that IMM has better capability in suppressing noise and diagnosing delay.

#### Simulation without noise

**Fig 12:**
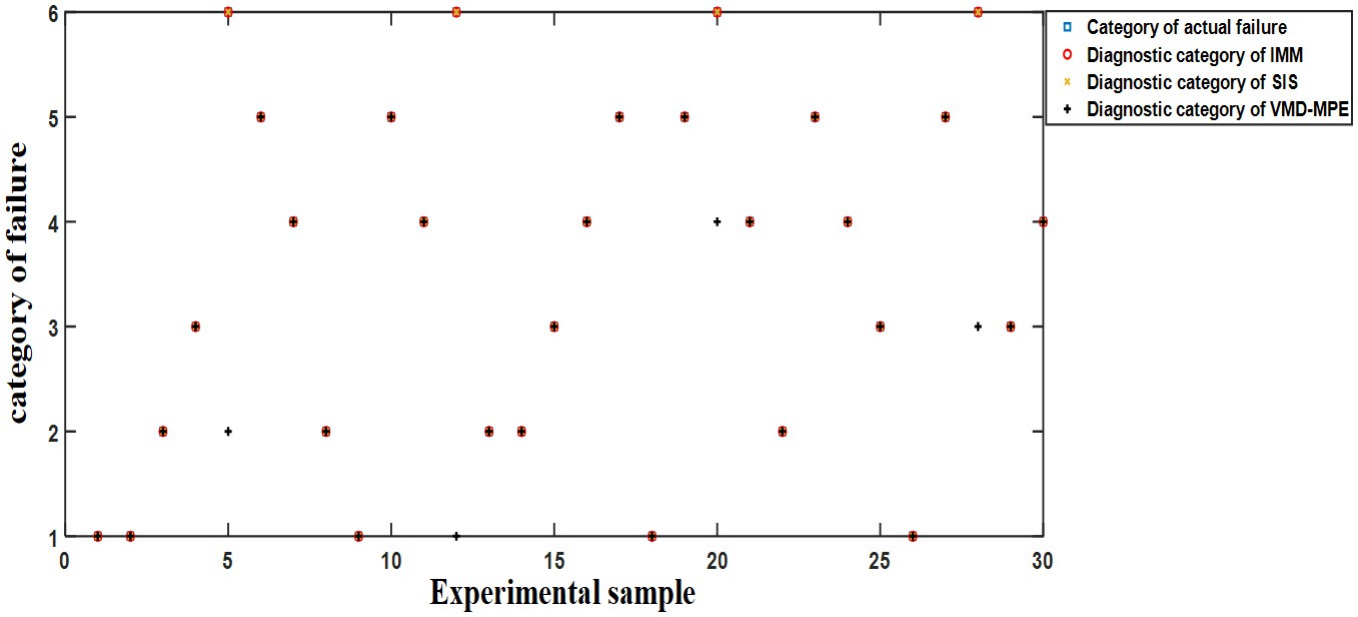
Fault diagnosis results of each method without noise.

Experiment: The SRM power converter is configured to work normally, with single-phase chopper tube short-circuits and open-circuit faults, single-phase position tube short-circuits and open-circuit faults, and multi-phase mixed faults, each with 20 groups each. Besides, 30 groups were randomly selected as experimental training samples. The category labels of normal operation, single-phase chopper tube short-circuits and open-circuit faults, single-phase position tube short-circuits and open-circuit faults, and multi-phase mixed faults were represented as “1”, “2”, “3”, “4”, “5”, “6”, respectively. The fault diagnosis results of each method without noise are provided in Fig 12.

Fig 12 implies that in the absence of noise, the fault diagnosis accuracy of IMM and SIS is 100%, while VMD-MPE cannot identify multiphase faults, and the diagnosis accuracy is only 86.67%.

#### Simulation with noise

**Fig 13:**
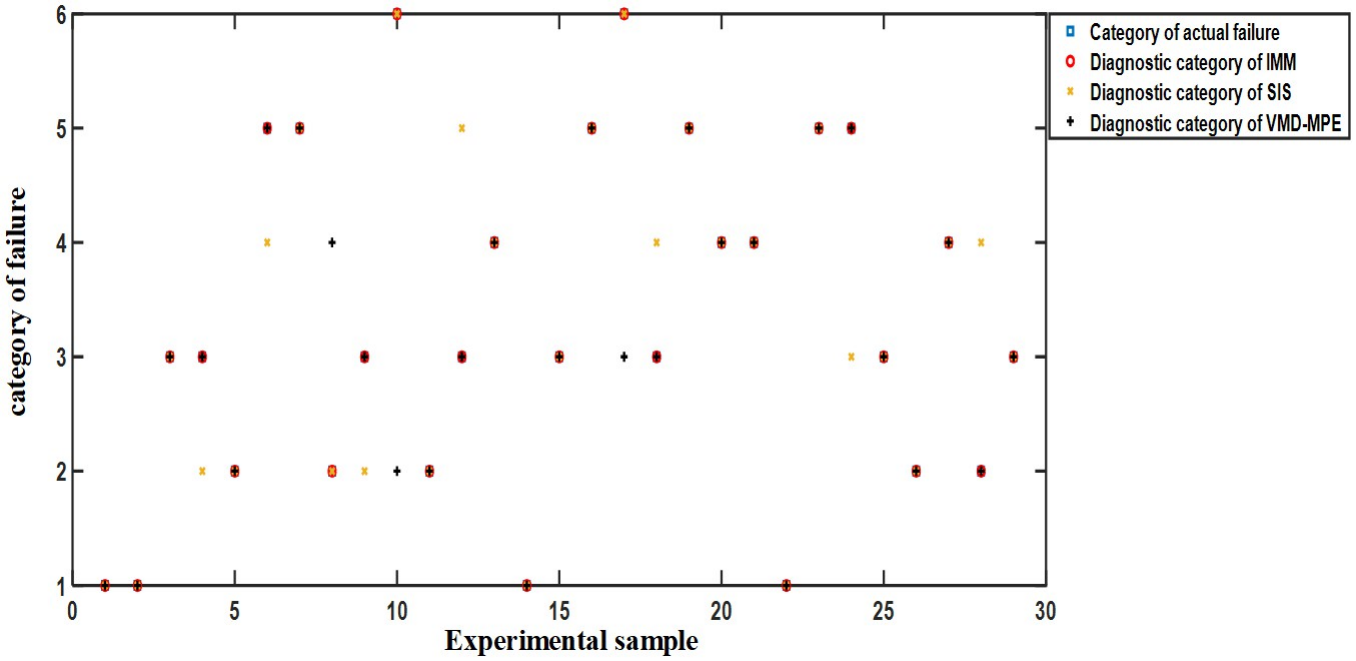
Fault diagnosis results of various methods in the case of noise.

Experiment: Same as Section 5.4.1, 30 groups of system states are randomly selected as experimental training samples, and different Gaussian white noises are added to the sampled signals of the SRM power converter. The fault diagnosis results of each method in the case of noise are illustrated in Fig 13.

Fig 13 demonstrates that in the case of noise, the fault diagnosis accuracy of IMM is 100%, while the fault diagnosis accuracy of SIS and VMD-MPE is 76.67% and 90%, respectively. Therefore, the noise immunity of IMM is better than that of SIS and VMD-MPE.

**Fig 14:**
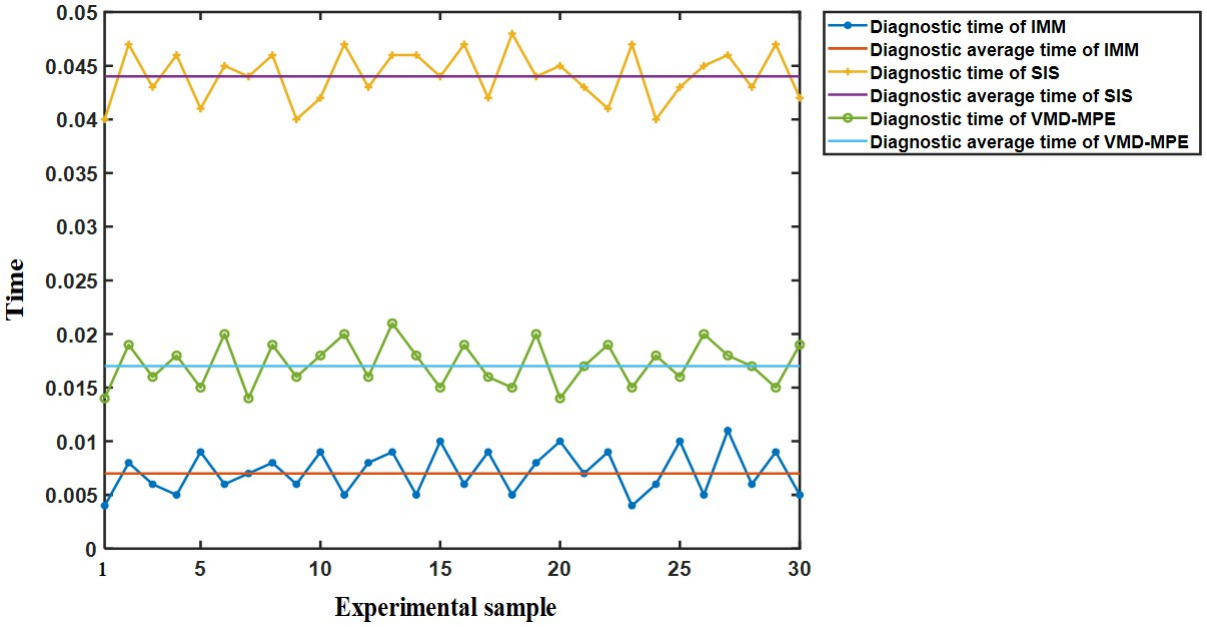
Fault diagnosis time curve of each method.

Additionally, the sampling time is set to 50 ms according to the data requirements of the method to compare the differences in the fault diagnosis delay of each method. The fault diagnosis time curve of each method is exhibited in Fig 14. Table 11 presents a comparison of fault diagnosis time.

**Table 11:** Comparison of fault diagnosis time.

Diagnosis method	Average time(s)	Maximum time(s)	Minimum time(s)
**IMM**	0.007	0.011	0.004
**SIS**	0.017	0.021	0.014
**VMD-MPE**	0.044	0.047	0.04

As revealed in Fig 14 and Table 11, the fault diagnosis time of IMM is the shortest, and the average time is 0.007 s. Therefore, IMM is superior to SIS and VMD-MPE in diagnostic delay.

## Experimental results and analysis

**Fig 15:**
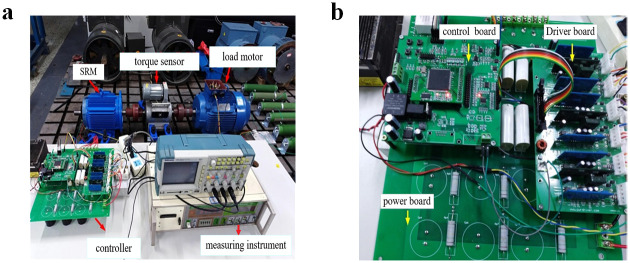
SRM experiment platform. (a) SRM test bench. (b) SRM control platform.

The experimental platform includes SRM, torque sensor, load motor, motor controller, and so on. The torque sensor is used to measure the torque and speed of the SRM, and the load motor uses a permanent magnet generator, which provides a continuously adjustable load for the SRM by adjusting the output current of the permanent magnet generator. The controller includes a control board, a driver board and a power board. The control board mainly includes DSP control chip, position detection circuit and voltage and current detection circuit. The driver board uses DA962 to form an integrated driver, and The power board is composed of IGBT power module to form SRM asymmetric power conversion circuit. The experimental test platform is shown in Fig 15. The equipment used in the experiment is shown in Table 12.

**Table 12:** Table of SRM experimental equipment.

The name of the devices	Parameters
**SRM**	6/4-pole, rated power is 3 kw, Rated voltage is 514 V, Rated speed is 3000 r/min
**Load motor**	*FZ*200 JY, Rated torque is 200 N ⋅ m, Exciting current is 18 A
**Torque sensor**	*CYB* − 803 S, Measuring range is 0 − ±50 N ⋅ m
**Adjustable DC Power Supply**	*AMETEKSCI* − 500/60 C, 0 − 500 V, 0 − 60 A
**DSP emulator**	*ICETEK* − 5100 USB
**Oscilloscope**	*TektronixTPS*2024, four channel
**Multimeter**	*FULKE*17 B

**Fig 16:**
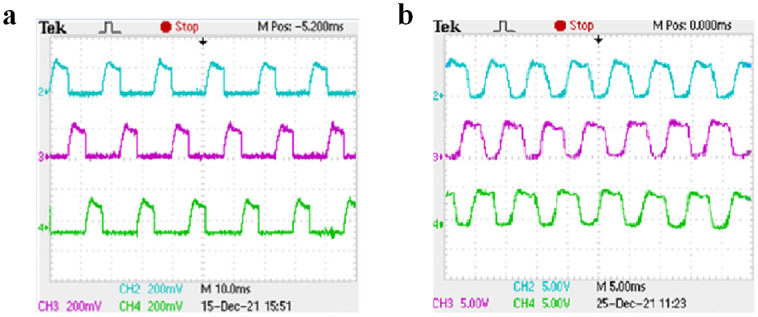
Three-phase current waveforms for normal operation. (a) The speed is 500 r/min. (b) The speed is 1000 r/min.

**Fig 17:**
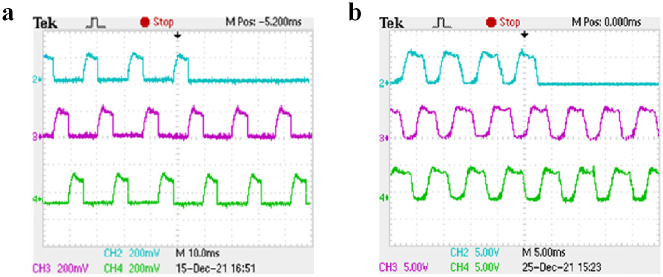
The open-circuit fault of A-phase SA1 experimental waveform. (a) The speed is 500 r/min. (b) The speed is 1000 r/min.

**Fig 18:**
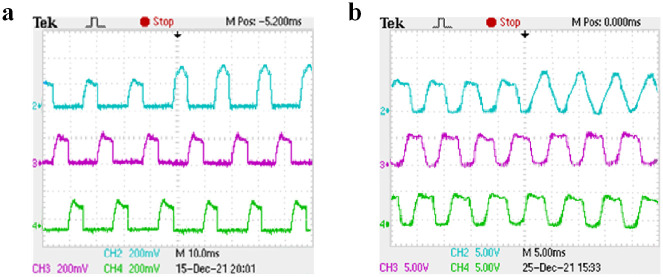
The short-circuit fault of A-phase SA1 experimental waveform. (a) The speed is 500 r/min. (b) The speed is 1000 r/min.

**Fig 19:**
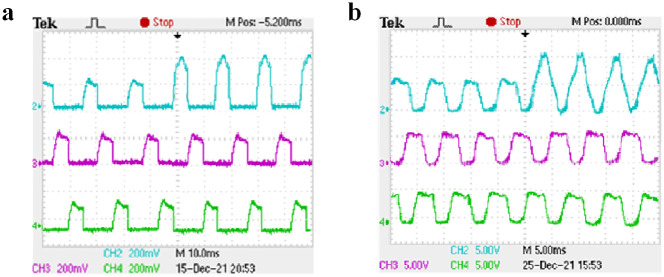
The short-circuit fault of A-phase SA2 experimental waveform. (a) The speed is 500 r/min. (b) The speed is 1000 r/min.

Under the condition of closed-loop speed control, the starting load of the motor is set to 10 N ⋅ m. Channels 2, 3, and 4 of the oscilloscope display the current waveforms of phases A, B, and C, respectively. Taking phase A as an example, when the rotating speed is set to 500 r/min and 1000 r/min and different switching tube faults are set, the three-phase current waveform under normal operation is shown in Fig 16, the current waveform before and after the open-circuit fault of A-phase SA1 is shown in Fig 17, the current waveform before and after the short-circuit fault of A-phase SA1 is shown in Fig 18, and the current waveform before and after the short-circuit fault of A-phase SA2 is shown in Fig 19.

As shown in Fig 16, when the motor is running at different speeds, the on-time of the three-phase current is different and the amplitude is the same. It can be seen from Fig 17 that when the A-phase SA1 occurred open-circuit fault, the A-phase current will drop to zero, the other two-phase current waveforms do not change, and the motor is in a phase-opening state. It can be seen from Figs 18 and 19 that when when the A-phase SA1 occurred short-circuit fault, the current amplitude will increases about 1.5 times of the original, and when the A-phase SA2 occurred short-circuit fault, the current amplitude will increase about 2 times and the current waveform of the other two phases does not change. The experimental results show that when different switch tube faults occur, the corresponding fault model probability will increase. And the IMM diagnosis method can quickly identify the occurrence of the fault and accurately locate the fault location.

In order to further prove the accuracy and effectiveness of the IMM diagnosis method, this paper sets up multiple groups of fault experimental test samples, and identify the faults by the three methods of IMM, SIS, and VMD-MPEThe diagnosis results are shown in Table 13.

**Table 13:** Diagnostic results of each method.

	Fault type	Number of samples tested	Diagnostic results			Comprehensive accuracy/%
			Normal	Open circuit	Short circuit	
**IMM**	**Normal**	30	30	—	—	100
	**Open circuit**	30	—	30	—	
	**Short circuit**	30	—	—	30	
**SIS**	**Normal**	30	28	1	1	85.66
	**Open circuit**	30	4	24	2	
	**Short circuit**	30	3	2	25	
**VMD-MPE**	**Normal**	30	29	1	—	92.22
	**Open circuit**	30	3	26	1	
	**Short circuit**	30	1	1	28	

It can be seen in Table 13 that when the SIS method is used, the recognition accuracy of open circuit faults is low, and the comprehensive recognition accuracy is 85.66%. When the VMD-MPE method is used, the accuracy of identifying normal and short-circuit operating states is high, but the accuracy of identifying the open-circuit fault is low, and the comprehensive identification accuracy is 92.22%. The identification accuracy of the IMM method is significantly higher than the other two methods, which can effectively improve the accuracy of the fault diagnosis of the SRM power converter.

## Conclusion

Given fault false alarm and fault control failure caused by the decrease of fault identification accuracy and fault delay of SRM power converter in complex working conditions, a method based on the IMM algorithm was proposed in this paper. The corresponding equivalent circuit models were established according to the different working states of the SRM power converter and the transition probability matrix was able to be corrected in real time; in the non-model transformation stage, a transition probability correction function was constructed with a difference in the n-th order based on model probability, making the matched model mix more historical information, suppressing the effect of noise and improving the estimation accuracy and diagnostic accuracy. However, in the model transformation stage, a model jump threshold was introduced in order to reduce the delay of the matched model. The matched model only carried little historical information, making the non-main diagonal elements of the transition probability matrix very small, improving the switching speed of model and reducing the diagnosis delay. Using STKF to estimate the state of the SRM power converter system, the direct detection and location of multiple faults in SRM power converter were realized according to the residual signal output by the Kalman filter. In the simulation analysis, when no failure was present, models 1, 2 and 3 were shown to alternately match with the system. Thus, it was inferred that no failure occurred, wherein the process of load and speed changes, the matching model probability reached 99%, while the maximum residual error of each sensor current did not exceed 0.035. When a single fault occurred, the matching model probability decreased by 1%, the current residual error of each sensor rose by 0.003 and remained around 0.023, and the maximum residual error was 0.038. The system was also able to accurately determine the type of fault based on the matching model, after which other faults occurred. The matching probability as well as the residual current of each sensor were shown to not greatly change and had a corresponding matching model that matched the current power converter state, thereby demonstrating the occurrence of multiple faults. In light of the above findings, when multiple faults occur at the same time, their occurrence can still be accurately determined by matching the model probability as well as the residual value of the current of each sensor, and separation of the faults can be achieved. Compared with other methods, the results show that when there was noise interference, the fault diagnosis accuracy of IMM was 100% and the average time of fault diagnosis was 0.007 s, and its anti-interference ability and diagnosis delay were better than SIS and VMD-MPE. In the experimental research, when the fault of the switch tube at different speeds occurred, the IMM diagnosis method can quickly identify the occurrence of the fault and accurately locate the fault location. The experimental results compared with SIS and VMD-MPE also showed that the identification accuracy of the IMM method was significantly higher than the other two methods. The results of simulation and experiment fully illustrate that the proposed IMM multi-fault diagnosis algorithm can still quickly and accurately estimate the operating state of the SRM power converter under complex operating conditions, detect multiple faults in real time, and perform fault separation. Therefore, the IMM algorithm may achieve the accurate detection and rapid separation of multiple fault information in SRM power converter.

## References

[1] Sun YX, Mu HW, Gao JH. Design and protection of the controller for SRD system. In: 2008 International Conference on Machine Learning and Cybernetics. vol. 4; 2008. p. 1943–1946.

[2] Kumar SS, Jayakumar J. Torque modeling of Switched Reluctance Motor using LSSVM-DE. Neurocomputing. 2016;211:117–128. doi: 10.1016/j.neucom.2016.02.076

[3] Belhadi M, Krebs G, Marchand C. Geometrical optimization of SRM on operating mode for automotive application. Electrical Engineering. 2018;100(1):303–310. doi: 10.1007/s00202-016-0504-0

[4] Kozuka S, Tanabe N, Asama J, Chiba A. Basic characteristics of 150,000r/min switched reluctance motor drive. In: 2008 IEEE Power and Energy Society General Meeting—Conversion and Delivery of Electrical Energy in the 21st Century; 2008. p. 1–4.

[5] Gopalakrishnan S, Omekanda AM, Lequesne B. Classification and remediation of electrical faults in the switched reluctance drive. IEEE Transactions on Industry Applications. 2006;42(2):479–486. doi: 10.1109/TIA.2006.870044

[6] L S, C H, Z H, Y D. On-line diagnosis method for power converter faults in switched reluctance motors. Proceedings of the Chinese Society for Electrical Engineering. 2010;30:63–70.

[7] Z J, X L, B D. Fault Diagnosis of Switched Reluctance Motor Power Converter Based on VMD-MPE. In: 2021 24th International Conference on Electrical Machines and Systems (ICEMS); 2021. p. 2546–2550.

[8] Bouchareb I, Bentounsi A, Lebaroud A. Fault detection and diagnosis in a set “inverter-switched reluctance motor” based on pattern recognition using Kalman filter prediction. International Journal of Applied Electromagnetics and Mechanics. 2014; p. 495–502. doi: 10.3233/JAE-141869

[9] Hossein T. Intern-turn Short-circuit Fault Detection in Switched Reluctance Motor Utilizing MCPT Test. International Journal of Applied Electromagnetics and Mechanics. 2014; p. 619–628.

[10] Pei X, Nie S, Chen Y, Kang Y. Open-Circuit Fault Diagnosis and Fault-Tolerant Strategies for Full-Bridge DC–DC Converters. IEEE Transactions on Power Electronics. 2012;27(5):2550–2565. doi: 10.1109/TPEL.2011.2173589

[11] Sun X, Tong X, Yin J. Fault Diagnosis for VSC-HVDC Using Wavelet Transform. In: 2012 Asia-Pacific Power and Energy Engineering Conference; 2012. p. 1–4.

[12] Gan C, Wu J, Yang S. Fault diagnosis of power converter for switched reluctance motor based on discrete degree analysis of wavelet packet energy. In: 2013 International Conference on Electrical Machines and Systems (ICEMS). vol. 34; 2013. p. 768–772.

[13] Peter WT, Yang Wx, Tam HY. Machine fault diagnosis through an effective exact wavelet analysis. Journal of Sound and Vibration. 2004;277:1005–1024. doi: 10.1016/j.jsv.2003.09.031

[14] Yan R, Gao RX, Chen X. Wavelets for fault diagnosis of rotary machines: A review with applications. Signal Processing. 2014;96:1–15. doi: 10.1016/j.sigpro.2013.04.015

[15] Syafi’i MHRA, Prasetyono E, Khafidli MK, Anggriawan DO, Tjahjono A. Real Time Series DC Arc Fault Detection Based on Fast Fourier Transform. In: 2018 International Electronics Symposium on Engineering Technology and Applications (IES-ETA); 2018. p. 25–30.

[16] Pandarakone SE, Masuko M, Mizuno Y, Nakamura H. Deep Neural Network Based Bearing Fault Diagnosis of Induction Motor Using Fast Fourier Transform Analysis. In: 2018 IEEE Energy Conversion Congress and Exposition (ECCE); 2018. p. 3214–3221.

[17] Lei Y, Lin J, He Z, Zuo MJ. A review on empirical mode decomposition in fault diagnosis of rotating machinery. Mechanical Systems and Signal Processing. 2013;35(1):108–126. doi: 10.1016/j.ymssp.2012.09.015

[18] Gao Q, Duan C, Fan H, Meng Q. Rotating machine fault diagnosis using empirical mode decomposition. Mechanical Systems and Signal Processing. 2008;22(5):1072–1081. doi: 10.1016/j.ymssp.2007.10.003

[19] Ruikun Y, Ruiqing M, Peng B. Application of HHT in SRM fault feature extraction. In: 2017 IEEE 2nd Advanced Information Technology, Electronic and Automation Control Conference (IAEAC); 2017. p. 57–63.

[20] Yan X, Liu Y, Zhang W, Jia M, Wang X. Research on a Novel Improved Adaptive Variational Mode Decomposition Method in Rotor Fault Diagnosis. Applied Sciences. 2020;10(5):1696. doi: 10.3390/app10051696

[21] Cui H, Guan Y, Chen H. Rolling Element Fault Diagnosis Based on VMD and Sensitivity MCKD. IEEE Access. 2021;9:120297–120308. doi: 10.1109/ACCESS.2021.3108972

[22] Li G, Li Y, Chen H, Deng W. Fractional-Order Controller for Course-Keeping of Underactuated Surface Vessels Based on Frequency Domain Specification and Improved Particle Swarm Optimization Algorithm. Applied Sciences. 2022;12(6):3139. doi: 10.3390/app12063139

[23] Deng W, Li Z, Li X, Chen H, Zhao H. Compound Fault Diagnosis Using Optimized MCKD and Sparse Representation for Rolling Bearings. IEEE Transactions on Instrumentation and Measurement. 2022;71:1–9. doi: 10.1109/TIM.2022.3159005

[24] Deng W, Zhang X, Zhou Y, Liu Y, Zhou X, Chen H, et al. An enhanced fast non-dominated solution sorting genetic algorithm for multi-objective problems. Information Sciences. 2022;585:441–453. doi: 10.1016/j.ins.2021.11.052

[25] Gan C, Wu J, Yang S, Hu Y, Cao W. Wavelet Packet Decomposition-Based Fault Diagnosis Scheme for SRM Drives With a Single Current Sensor. IEEE Transactions on Energy Conversion. 2016;31(1):303–313. doi: 10.1109/TEC.2015.2476835

[26] Ro HS, Kim DH, Jeong HG, Lee KB. Tolerant Control for Power Transistor Faults in Switched Reluctance Motor Drives. IEEE Transactions on Industry Applications. 2015;51(4):3187–3197. doi: 10.1109/TIA.2015.2411662

[27] Diallo D, Benbouzid MEH, Hamad D, Pierre X. Fault detection and diagnosis in an induction Machine drive: a pattern recognition approach based on concordia stator mean current vector. IEEE Transactions on Energy Conversion. 2005;20(3):512–519. doi: 10.1109/TEC.2005.847961

[28] Peng W, Gyselinck JJC, Ahn JW, Lee DH. Minimal Current Sensing Strategy for Switched Reluctance Machine Control With Enhanced Fault-Detection Capability. IEEE Transactions on Industry Applications. 2019;55(4):3725–3735. doi: 10.1109/TIA.2019.2904433

[29] Gan C, Wu J, Yang S, Hu Y, Cao W, Si J. Fault diagnosis scheme for open-circuit faults in switched reluctance motor drives using fast Fourier transform algorithm with bus current detection. IET Power Electronics. 2016;9(1):20–30. doi: 10.1049/iet-pel.2014.0945

[30] L Y, J K. Fault Diagnosis of Switched Reluctance Motor Power Converter Based on State Inverse Solution. Journal of Central South University (Natural Science Edition). 2021;52(4):1185–1196.

[31] Jingwen Z, Lixin X, Dunxin B. Fault Diagnosis of Switched Reluctance Motor Power Converter Based on VMD-MPE. In: 2021 24th International Conference on Electrical Machines and Systems (ICEMS); 2021. p. 2546–2550.

[32] Rong Li X, Jilkov VP. Survey of maneuvering target tracking. Part V. Multiple-model methods. IEEE Transactions on Aerospace and Electronic Systems. 2005;41(4):1255–1321. doi: 10.1109/TAES.2005.1561886

[33] Seah CE, Hwang I. Algorithm for Performance Analysis of the IMM Algorithm. IEEE Transactions on Aerospace and Electronic Systems. 2011;47(2):1114–1124. doi: 10.1109/TAES.2011.5751246

[34] Gadsden SA, Song Y, Habibi SR. Novel Model-Based Estimators for the Purposes of Fault Detection and Diagnosis. IEEE/ASME Transactions on Mechatronics. 2013;18(4):1237–1249. doi: 10.1109/TMECH.2013.2253616

[35] Vasuhi S, Vaidehi V. Target tracking using Interactive Multiple Model for Wireless Sensor Network. Information Fusion. 2016;27:41–53. doi: 10.1016/j.inffus.2015.05.004

[36] Zhang K, Jiang B, Chen F. Multiple-Model-Based Diagnosis of Multiple Faults With High-Speed Train Applications Using Second-Level Adaptation. IEEE Transactions on Industrial Electronics. 2021;68(7):6257–6266. doi: 10.1109/TIE.2020.2994867

[37] Hayashi Y, Tsunashima H, Marumo Y. Fault Detection of Railway Vehicles Using Multiple Model Approach. In: 2006 SICE-ICASE International Joint Conference; 2006. p. 2812–2817.

[38] Wang Y, Meng D, Li R, Zhou Y, Zhang X. Multi-Fault Diagnosis of Interacting Multiple Model Batteries Based on Low Inertia Noise Reduction. IEEE Access. 2021;9:18465–18480. doi: 10.1109/ACCESS.2021.3051986

[39] Miller TJE. Electronic control of switched reluctance machines. Elsevier; 2001.

[40] Chen H, Lu S. Fault Diagnosis Digital Method for Power Transistors in Power Converters of Switched Reluctance Motors. IEEE Transactions on Industrial Electronics. 2013;60(2):749–763. doi: 10.1109/TIE.2012.2207661

[41] Gameiro NS, Marques Cardoso AJ. A New Method for Power Converter Fault Diagnosis in SRM Drives. IEEE Transactions on Industry Applications. 2012;48(2):653–662. doi: 10.1109/TIA.2011.2180876

[42] Cork L, Walker R. Sensor Fault Detection for UAVs using a Nonlinear Dynamic Model and the IMM-UKF Algorithm. In: 2007 Information, Decision and Control; 2007. p. 230–235.

[43] Hsiao R, Tam YC, Schultz T. Generalized Baum-Welch algorithm for discriminative training on large vocabulary continuous speech recognition system. In: 2009 IEEE International Conference on Acoustics, Speech and Signal Processing; 2009. p. 3769–3772.

[44] Xiong H, Tang J, Xu H, Zhang W, Du Z. A Robust Single GPS Navigation and Positioning Algorithm Based on Strong Tracking Filtering. IEEE Sensors Journal. 2018;18(1):290–298. doi: 10.1109/JSEN.2017.2767066

[45] Zhu B, He H. Integrated navigation for doppler velocity log aided strapdown inertial navigation system based on robust IMM algorithm. Optik. 2020;217:164871. doi: 10.1016/j.ijleo.2020.164871

[46] Tian Z, Li Y, Cen M, Zhu H. Multi-Vehicle Tracking Using an Environment Interaction Potential Force Model. IEEE Sensors Journal. 2020;20(20):12282–12294. doi: 10.1109/JSEN.2020.2999095

[47] Sun L. Research and application of the interacting multiple model algorithm with adaptive transition probability matrix. Xi’an University of Technology. 2019.

